# Single-Cell Sequencing of Immune Cells in Human Aortic Dissection Tissue Provides Insights Into Immune Cell Heterogeneity

**DOI:** 10.3389/fcvm.2022.791875

**Published:** 2022-03-31

**Authors:** Yifan Liu, Lingwei Zou, Hanfei Tang, Jie Li, Hao Liu, Xiaolang Jiang, Baohong Jiang, Zhihui Dong, Weiguo Fu

**Affiliations:** ^1^Department of Vascular Surgery, Institute of Vascular Surgery, Zhongshan Hospital, Fudan University, Shanghai, China; ^2^National Clinical Research Center for Interventional Medicine, Shanghai, China; ^3^Shanghai Institute of Materia Medica, Chinese Academy of Sciences, Shanghai, China

**Keywords:** single-cell RNA sequencing, aortic dissection, immune cell heterogeneity, T cells, macrophages, leukocyte

## Abstract

**Background:**

Inflammation plays an important role in the progression of sporadic aortic dissection (AD). Immune cells, especially macrophages, infiltrate the aorta and secrete inflammatory cytokines and matrix metalloproteinases to cause degradation of the extracellular matrix, thereby contributing to the pathogenesis of AD. However, the cellular heterogeneity within these immune cells has not been fully characterized.

**Methods:**

We used single-cell RNA sequencing to profile the transcriptomes of all immune cells in AD tissue and normal aorta. Using magnetic-activated cell sorting gating on CD45, we obtained a higher resolution identification of the immune cell subsets in the aorta.

**Results:**

We observed significant differences in the proportion of major immune cell subpopulations between AD and normal aorta tissues. Macrophages accounted for a higher percentage in the normal aorta, while the proportions of T cells, B cells and natural killer (NK) cells were all increased in AD tissues. Macrophage clusters that expanded in AD tissues originated primarily from circulating monocytes and expressed genes encoding proinflammatory cytokines and molecules involved in tissue repair. T and NK cells in AD tissues exhibited enhanced cytotoxic properties. A cluster of CD4^+^ T cells that had expanded in AD tissues was Th17-like and might contribute to the pathogenesis of AD. Cell–cell interaction analysis highlighted the increased communication between macrophages and T cells, which primarily regulated the costimulation of T cells.

**Conclusions:**

Our study provides a comprehensive characterization of immune cells in the dissected aorta with an emphasis on the role of macrophages and T cells. The information from our study improves our understanding of immune mechanisms in AD formation and helps to identify additional useful targets for early diagnosis or therapy of AD.

## Introduction

Aortic dissection (AD) is a life-threatening cardiovascular emergency characterized by destruction of the tunica media and separation of the aortic wall (1). Aortic inflammation is one of the primary features of AD. The infiltrated immune cells in the tunica media and adventitia can cause increased oxidative stress and expression of inflammatory factors and matrix metalloproteinases (MMPs), thereby contributing to vascular smooth muscle cell (VSMC) apoptosis and aortic remodeling, playing an important role in the pathogenesis of AD (2).

The infiltrating immune cells consist of multiple cell types, including neutrophils, mast cells, macrophages, and lymphocytes. It has been reported that aortic immune cells are highly heterogeneous, with different cellular states and functions in each population, playing different roles during AD progression (3). The presence of infiltrated macrophages is often linked to elevated MMP expression, elastic fiber degeneration and VSMC apoptosis, while different subtypes of T lymphocytes may function differently in AD. In addition, these inflammatory cells, especially macrophages, can have multiple origins (4). Although they are mostly thought to be recruited from the circulation, evidence suggests that local proliferation is an important way to refill aortic macrophages after replenishment, and VSMCs can undergo phenotypic changes and switch to a macrophage-like phenotype (4). How these resident or SMC-derived macrophages functionally differ from recruited macrophages and potentially contribute to AD is unknown. Hence, the global and systematic investigation of heterogeneous immune cell states, functions and origins is important to improve our understanding of how inflammation contributes to AD pathogenesis.

Single-cell RNA sequencing (scRNA-seq) is a newly developed tool that characterizes the transcription of genes in individual cells in an unbiased manner without prior knowledge of genes or proteins of interest and can be used to group cell populations, reveal their markers and functional states, and investigate their relationship with disease (5). Recently, studies using scRNA-seq to characterize the cell subsets of the aorta in a mouse model of Marfan syndrome or infrarenal abdominal aneurysm and patients with ascending thoracic aortic aneurysm have emerged (6–9). However, scRNA-seq studies on human AD tissues are lacking. In addition, due to the vast number of non-immune cells in the aorta, the investigation of less abundant immune cell subsets could be hindered by lower resolution (10–12).

To concentrate on inflammation in the aorta, we used leukocyte common antigen (CD45) to sort immune cells by magnetic-activated cell sorting (MACS), and all subsequent scRNA-seq analyses were performed on cells gated on CD45 (10–12). Here, we compare the transcriptional profiles of immune cells in AD tissues and normal aortas. Our results revealed that inflammation in AD tissues involves not only macrophages but also adaptive immune cells, especially T cells. Overall, we provide a comprehensive description of immune cells in AD tissues that may help to further explore the immune mechanism of AD and the identification of new therapeutic targets.

## Materials and Methods

### Tissue Sample Collection

Aortic dissection tissues were collected from 3 patients with acute Stanford type A aortic dissection, and control aortic tissues were obtained from 2 heart transplant donors. This study was approved by the ethical committee of Zhongshan Hospital, Fudan University, China. Written informed consent was provided by all participants or their legal representatives.

### Preparation of Single-Cell Suspensions and MACS Sorting of Immune Cells

Samples were washed with calcium-free and magnesium-free phosphate-buffered saline and then separated into thin layers and cut into small pieces on ice. The tissues were then digested using an enzyme cocktail, which consisted of 3 mg/ml collagenase type I (Sigma–Aldrich), 0.156 mg/ml collagenase type XI (Sigma–Aldrich), 0.25 mg/ml soybean trypsin inhibitor (Worthington), 0.1875 mg/ml lyophilized elastase (Worthington), 0.24 mg/ml hyaluronidase type I (Sigma–Aldrich) and 60 U/ml DNase I (Sigma–Aldrich) (6, 13). Tissues were digested in a 37°C water bath for 30 min, the supernatants were collected, with Dulbecco's modified Eagle's medium (DMEM) and 10% fetal bovine serum added to stop the reaction. Fresh enzyme cocktail was then added to the digested tissues for a second round of digestion. The digestion process was repeated 3–4 times. Then, the erythrocytes were removed via ACK lysis buffer (Lonza) treatment for 5 min. The CD45^+^ cells were then purified using a magnetic cell sorting system (Miltenyi Biotec) according to the manufacturer's instructions. Following centrifugation (300 g, 4°C, and 5 min), the cell pellet was collected. Finally, 10 μl of suspension was counted under an inverted microscope using a hemocytometer. Trypan blue was used to quantify live cells.

### Single-Cell RNA Sequencing

The single-cell RNA-seq libraries were constructed using a Chromium Single Cell 3' Reagent Kit, version 2, according to the manufacturer's instructions. Single-cell suspensions were loaded onto the Chromium Single Cell Controller Instrument (10 × Genomics, Pleasanton, CA, USA) to generate single-cell gel beads in emulsions (GEMs). After the cells in the GEM were lysed, reverse transcription reactions using barcoded full-length cDNA were performed, and the cDNA was then amplified using PCR with appropriate cycles. The amplified cDNA was then fragmented, end-repaired, A-tailed, index adaptor ligated, and subjected to library amplification. The constructed libraries were sequenced on the Illumina NovaSeq6000 system.

### Single-Cell RNA-Seq Data Processing

The CellRanger software pipeline (version 3.1.0, 10x Genomics) was used to demultiplex cellular barcodes, map reads to the genome and transcriptome using the STAR aligner, and downsample reads as required to generate normalized aggregate data across samples, producing a matrix of gene counts vs. cells. Global mapping statistics, such as the estimated number of cells, mean reads per cell and mean genes per cell for each sample, are reported in Table S1. The unique molecular identifier (UMI) count matrix was processed in R using the Seurat R package (version 3.1.2) (14). To remove low-quality cells and likely multiplet captures, we applied a criterion to filter out cells with UMI/gene numbers out of the limit of mean value ± 2-fold of standard deviations assuming a Gaussian distribution of each cell's UMI/gene numbers. We further discarded low-quality cells where >20% of the counts belonged to mitochondrial genes, following visual inspection of the distribution of cells by the fraction of mitochondrial genes expressed. After applying these QC criteria, 31,755 immune cells were included in downstream analyses. The “NormalizeData” function in Seurat was used for library size normalization and to obtain the normalized count (14). Specifically, the “LogNormalize” method normalized the gene expression measurements for each cell by the total expression, multiplied by a scaling factor (10,000 by default), and the results were log transformed.

Identification of the most variable genes across single cells was performed using the method described in Macosko et al. (15). The FindVariableGenes function (mean.function = FastExpMean, dispersion.function = FastLogVMR) in Seurat was used to select the most variable genes. Removal of the batch effects in scRNA-seq data was achieved by performing the mutual nearest neighbors (MNN) using the R package batchelor (16). Graph-based clustering of cells was performed based on their gene expression profile using the FindClusters function in Seurat (14). We relied on biological background knowledge and adjusted different parameters to obtain a relatively appropriate number of clusters. The selection of clustering parameters primarily depended on the complexity of the target cells. A 2-dimensional uniform manifold approximation and projection (UMAP) algorithm was applied to visualize cells using the RunUMAP function in Seurat. The FindAllMarkers function in Seurat (test.use = bimod) was used to identify marker genes of each cluster (14). The FindAllMarkers function identifies the positive markers for a given cluster of cells compared to all other cells. Differentially expressed genes (DEGs) were identified using the FindMarkers function (test.use = MAST) (14). The threshold for significantly differential expression was set as a *P*-value <0.05 and |log2foldchange| > 0.58. The GO enrichment and KEGG pathway enrichment analyses of DEGs were performed using R based on the hypergeometric distribution.

Gene set variation analysis (GSVA) was used to assign pathway activity estimates to cells of interest (17). The GSVA package (version 1.30.0) with standard settings was adopted. The LIMMA package (version 3.38.3) was used to calculate the differences in pathway activities scored on each cell.

The trajectory analysis was performed using the Monocle2 package (18). The raw count was imported into Monocle2, and ordered genes (qval <0.01) that could be informative in the ordering of cells along the pseudotime trajectory were selected using the differentialGeneTest function. After dimensional reduction clustering analysis, the inferred trajectory was acquired using the orderCells function with default parameters. The expression of selected genes was plotted using the plot_genes_in_pseudotime function.

The TFs (transcription factors) of different cell clusters in the groups were analyzed using the motifs database for RcisTarget and GRNboost (SCENIC version 1.1.2.2, which corresponds to RcisTarget 1.2.1 and AUCell 1.4.1) using default parameters (19).

The cell–cell communication analysis between different cell types was represented by the identification of biologically relevant ligand–receptor interactions from single-cell transcriptomics (scRNAseq) data using CellPhoneDB (version 2.0.0) (20). A ligand or a receptor was defined as “expressed” in a particular cell type if 10% of the cells of that type had non-zero read counts for the ligand/receptor encoding gene. The networks of cell–cell communication between any two cell types were defined first, and then the ligand–receptor interactions between selected cell types were displayed on a dot plot. The R packages Igraph and Circlize were used to display the cell–cell communication networks.

### Immunofluorescence

We adopted multiplexed immunofluorescence (mIF) to validate the primary findings of scRNA-seq. Formalin-fixed paraffin-embedded tissue sections of dissected and normal human ascending aortas were cut into 4 μm serial sections. A five-plex mIF assay was performed to validate the overall percent of each immune cell subtype (c-Kit for mast cells, CD66b for granulocytes, CD20 for B cells, CD68 for macrophages, and CD3 for T cells) present in the control and dissected aortas. An Opal Polaris 7-color Automation IHC Detection Kit (NEL871001KT, Akoya Biosciences, Marlborough, Massachusetts, USA) was used for mIF. Sections were prepared according to the instructions provided by the manufacturers. Briefly, sections were dewaxed and then underwent renaturation with sodium citrate or EDTA repair solution. Blocking buffer (Akoya) was used to block for 10 min. Then, sections were incubated with primary antibodies (at different dilutions and different incubation times for each marker) at room temperature, followed by incubation with Akoya's Opal Polymer anti-mouse and -rabbit HRP secondary antibodies for 10 min. The TSA fluor-marker pairings, staining order and other experimental conditions for the five-plex mIF assay are shown in Table S2.

Multispectral images were acquired using the Vectra Polaris Automated Quantitative Pathology Imaging System. All images were processed and analyzed using inForm software (V.2.6.0, Akoya Biosciences) for semiautomatic spectral unmixing, tissue identification, cell segmentation, cell classification, and quantification of expression intensity (21). After processing all images, the data were exported for further analysis of cell densities.

### Statistics

Continuous variables are presented as the mean ± standard deviation (SD), and categorical variables are presented as *n* (%). The χ^2^ or Fisher's exact test was applied to compare differences between categorical variables. All statistical analyses were performed using SPSS (version 20.0, Chicago, IL, USA) and R (Version 4.0.5, https://www.r-project.org). The Beanplot R package was used to generate violin plots, with the data distribution bandwidth evaluated using kernel density estimation. A two-tailed *P*-value < 0.05 was considered statistically significant.

## Results

### ScRNA-Seq Analysis of Human Sporadic Ascending Aortic Dissection-Associated Immune Cell Populations

**Figure 1:**
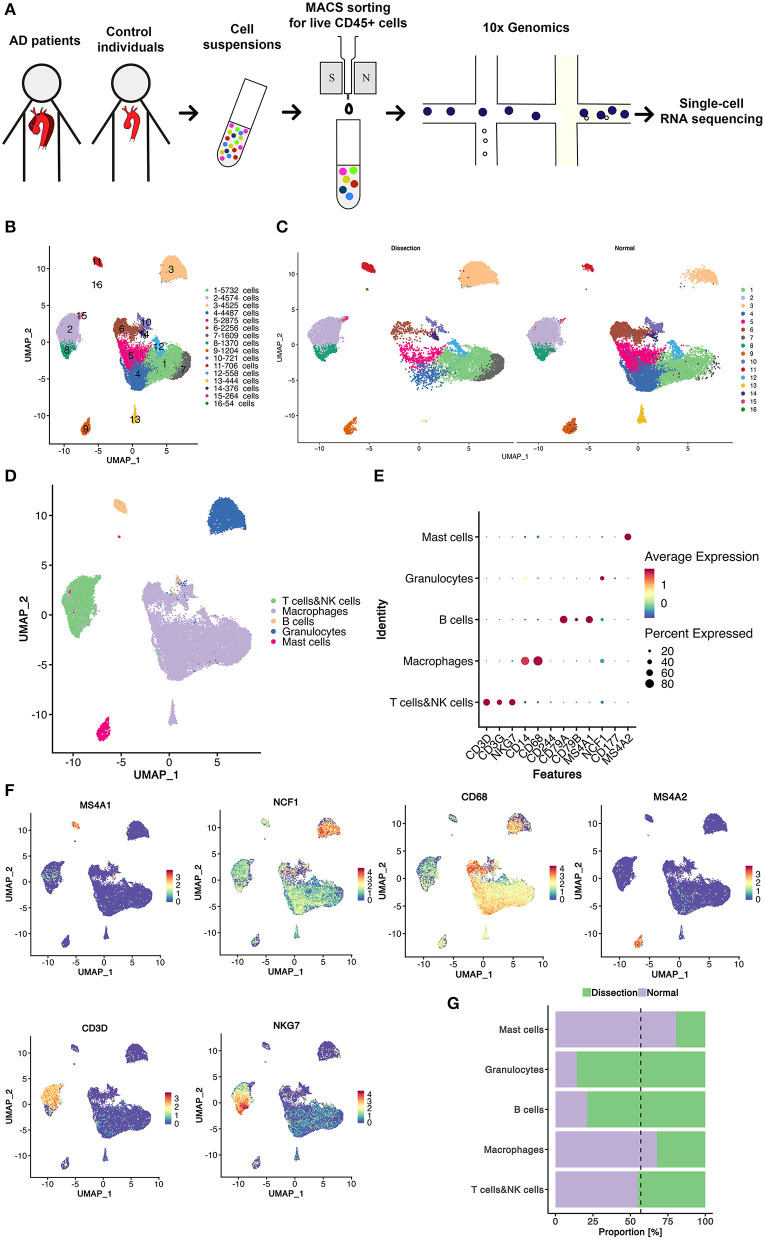
Sixteen clusters of immune cells identified using scRNA-seq analysis of human thoracic aortic dissection tissues. **(A)** Experimental approach of the experimental setup. Thoracic aortic dissection tissues were collected from 3 patients with sporadic type A aortic dissection, and normal aortas were collected from 2 heart transplant donors. Samples were digested, and live CD45^+^ cells were sorted by MACS and loaded for scRNA-seq. **(B)** The UMAP plot representation of 31,755 CD45^+^ immune cells colored by cluster. A total of 16 different clusters was identified after unsupervised clustering. **(C)** The sample origin of the cells. **(D)** The 5 major cell types identified by their marker genes. **(E,F)** Expression of marker genes for the different cell types. **(G)** The proportions of cells from the AD and NA groups in each cell type. The dashed line represents the proportion of cells from the AD group to all analyzed cells. MACS, magnetic-activated cell sorting; scRNA-seq, single-cell RNA-sequencing; UMAP, Uniform Manifold Approximation and Projection for Dimension Reduction; AD, aortic dissection; NA, normal aorta.

We obtained non-diseased ascending aortic wall tissue from 2 heart transplant donors and ascending aortic dissection tissues from 3 patients with sporadic type A aortic dissection. The clinical information of these patients is summarized in Table 1. MACS was used to sort CD45^+^ leukocytes. After quality filtering, viable CD45^+^ cells were subjected to scRNA-seq using a 10x Genomics-based single-tube protocol (Figure 1A). The distribution of the percentage of mitochondrial counts, nGene, and nUMI as well as the expression level of genes encoding hemoglobin constituents (*HBA1* and *HBA2*) are shown in Figure S1.

**Table 1:** Patient Information for Ascending Aortic Samples (*n* = 5).

**Variable**	**AD1**	**AD2**	**AD3**	**Control1**	**Control2**
Sex	M	M	M	M	M
Ethnicity	Chinese	Chinese	Chinese	Chinese	Chinese
Race	Asian	Asian	Asian	Asian	Asian
Age, y	44	54	43	24	35
Diagnosis and comments	TAAD	TAAD	TAAD	Heart transplant donor	Heart transplant donor
Aortic diameter, cm	4.2	4.7	4.2	NA	NA
Smoking status	Yes	Yes	Yes	No	No
Diabetes mellitus	No	No	No	No	No
Hypertension	Yes	Yes	Yes	No	No
Chronic obstructive pulmonary disease	No	No	No	No	No
Aortic valve regurgitation	No	Yes	No	No	No
Bicuspid aortic valve	No	No	No	No	No
Reoperation	No	No	No	No	No

*TAAD, type A aortic dissection; and NA, not available*.

After quality control and exclusion of non-immune cells and doublets, 31,755 immune cells were enrolled for further analysis. Of these cells, 13,680 cells (43.1%) were derived from AD tissues, and 18,075 cells (56.9%) were derived from normal non-diseased ascending aortas (NA group). These cells were classified into 16 clusters (Figure 1B). The UMAP plot revealed separation of cells between the AD and NA groups, indicating a potential difference in the distribution of leukocytes between the two groups (Figure 1C).

Based on the marker genes, we identified these immune cells as 5 cell types (Figure 1D), B cells (2.6%); granulocytes (14.3%); myeloid cells, including monocytes, macrophages, and dendritic cells (DCs) (59.5%); T cells and natural killer (NK) cells (19.4%) and mast cells (4.1%) (Figures 1E,F). The composition of each cell type exhibited a significantly different distribution between the AD and NA groups (Figure 1G). The granulocytes were expanded in AD tissues, while the proportion of myeloid cells in AD tissues was smaller compared to that in normal aortas. The proportions of T and NK cells in the two groups were basically the same. The B cells appeared to expand in the AD group, while mast cells were relatively more abundant in the normal aorta. However, this could be because these two cell types were inherently less abundant, so small changes in cell numbers would result in larger percent changes.

The top marker gene heatmap shows the unique expression profile of each cluster (Figure S2). Overall, this analysis demonstrated the presence of a complex immune cellular ecosystem, and we focused on several important cell subclusters to explore their functions.

### Macrophages Originating From Peripheral Circulation Contribute to AD Primarily by Secreting Proinflammatory Cytokines

**Figure 2:**
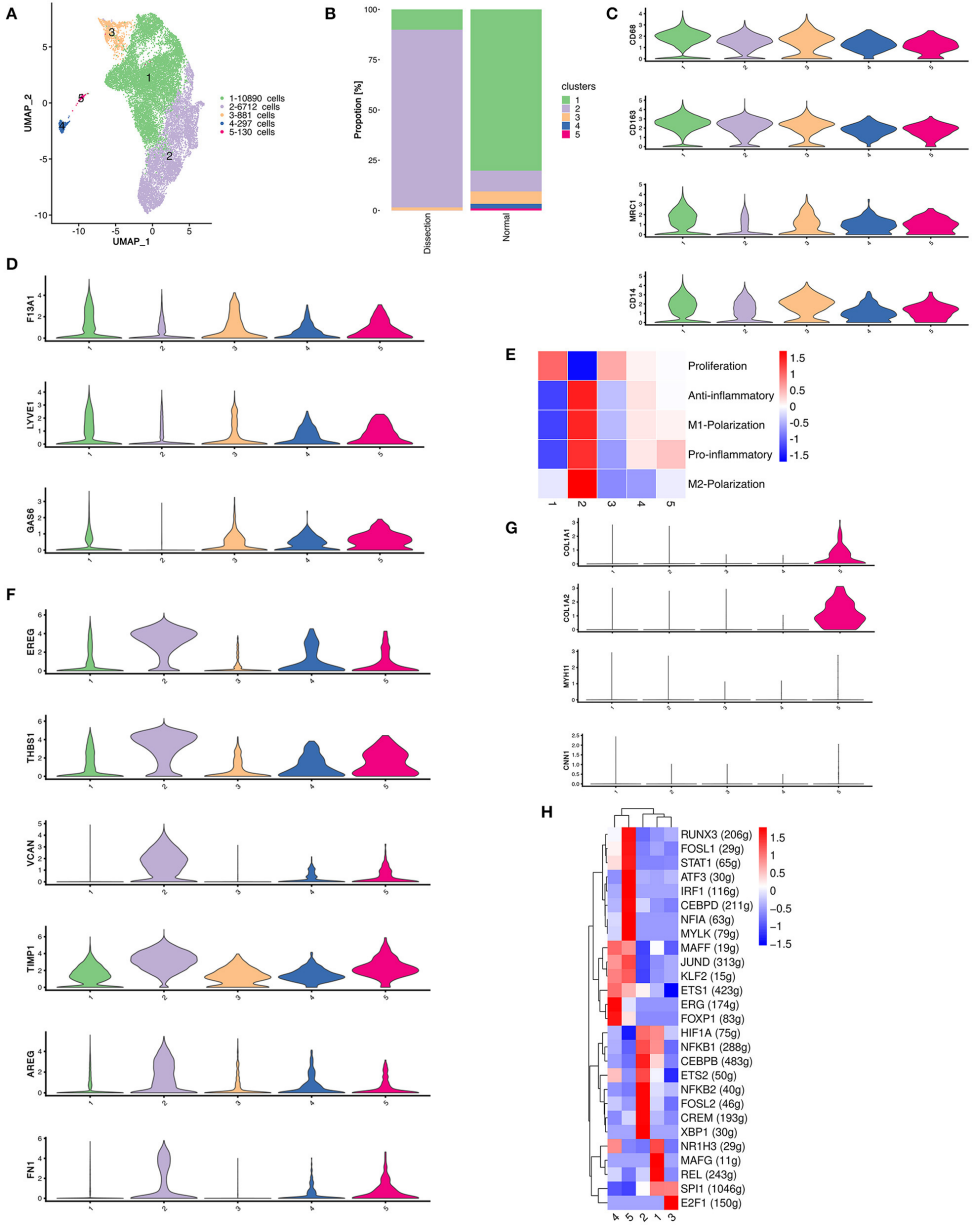
The heterogeneity of macrophages between normal and dissected aorta tissues. **(A)** A UMAP plot of 18,910 myeloid cells reclustered into 5 clusters. **(B)** The sample origin of myeloid cells showing differences in the distribution of cell clusters between the two groups. **(C)** Violin plots displaying the expression of marker genes in macrophages and dendritic cells. **(D)** Violin plots displaying the expression of marker genes of aortic resident macrophages. **(E)** Heatmap displaying the scores of proinflammatory, anti-inflammatory, M1 polarization, M2 polarization, and proliferation in each cluster. **(F)** Violin plots displaying the expression of TIMPs and other genes involved in ECM repair. **(G)** The violinplot of genes involved in collagen organization and markers for SMCs. **(H)** SCENIC analysis of the top differentially expressed transcription factors among different clusters of myeloid cells. UMAP, uniform manifold approximation and projection for dimension reduction; ECM, extracellular matrix; TIMPs, tissue inhibitors of metalloproteinases; SCENIC, single-cell regulatory network inference and clustering.

In total, 18,910 myeloid cells were detected in this study, which were further divided into 5 clusters (Figure 2A). Differences in the distribution of cell clusters were observed between the two groups (Figure 2B). Among them, only cluster 2 had a higher proportion in AD samples. All 5 clusters of myeloid cells expressed high levels of macrophage/monocyte markers (*CD68, CD163, MRCI, CD14*) (Figure 2C). Cluster 4 also expressed dendritic cell markers (*NRP1, IL3RA*) and was defined as dendritic cells (DCs) (22).

We then attempted to determine the origins of these clusters using several markers of resident macrophages, including *F13A1, LYVE1*, and *GAS6* (Figure 2D) (10). These genes were least expressed in cluster 2, indicating that the majority of the expanded macrophage clusters in AD samples originated from the circulation rather than from the proliferation of resident macrophages.

Macrophages with different polarization statuses can have different roles in the pathogenesis of aortic dissection (23). However, we could not clearly distinguish between M1- and M2-polarized macrophages using well-defined M1/M2 markers, such as *MRC1* and *CD163*, since they were both expressed in almost all myeloid clusters (Figure 2C). Therefore, we calculated the M1 and M2 polarization, pro- and anti-inflammatory and proliferation scores of all myeloid clusters using reported gene sets (Figure 2E) (24). There were no clusters strictly resembling typical M1/M2 polarized macrophages. Cluster 2 highly expressed both M1 and M2 markers but acquired high pro- and anti-inflammatory scores concurrently. Hence, we attempted to further illustrate the function of each cluster by their marker genes and enrichment analysis using GSVA (Figures S3–S5).

We first concentrated on cluster 2 myeloid cells that were expanded in the AD samples. The heatmap of the top 20 marker genes selected based on the average log (fold change) for the 5 myeloid cell clusters showed that in addition to proinflammatory molecules encoding genes including *CXCL5, CCL20, CXCL1, IL1B, and PTGS2*, cluster 2 myeloid cells also highly expressed genes involved in ECM repair rather than degradation, including *VCAN, EREG, AREG, TIMP1*, and *FN1* (Figure 2F and Figure S3) (6). Pathway enrichment analysis revealed that cluster 2 myeloid cells were involved in the IL17 signaling pathway, TNF signaling pathway, JAK–STAT signaling pathway and HIF−1 signaling pathway, which could be involved in the activation of macrophages or the secretion of proinflammatory cytokines (25, 26). In addition, enrichment of the ECM–receptor interaction pathway was also found in cluster 2 myeloid cells, consistent with the upregulation of ECM repair genes (Figure S4).

The remaining 4 myeloid cell clusters accounted for a higher proportion in control samples. The heatmap of the top marker genes showed that cluster 1 myeloid cells were primarily resident macrophages (*F13A1*). The highly expressed genes involved in lipid metabolism (*APOE, PLTP, APOC1, LPL*) as well as the enrichment of the linoleic acid metabolism pathway, cholesterol metabolism pathway and lipoic acid metabolism both indicate that cluster 1 myeloid cells have important functions in lipid metabolism. In addition, the upregulation of *HMOX1* also demonstrated that this cluster of myeloid cells may be involved in the removal of senescent or dead red blood cells in the aortic wall. The proportion of cluster 3 was relatively small in both the AD and control groups. The heatmap of the top marker genes showed that the cell proliferation marker *MKI67* was highly expressed in cluster 3 myeloid cells. Other top marker genes primarily encoded constitutive proteins of microtubules or histones. These results indicate that cluster 3 myeloid cells are primarily proliferative in nature.

Cluster 4 was defined as DCs. The heatmap of the top marker genes revealed elevated expression of the chemokine receptor *ACKR1*, a molecule that has a role in cell adhesion, including *SELE, POSTN*, and the secreted enzymes *ADAMTS9* and *ADAMTS1*. Cluster 5 myeloid cells were related to connective tissue development and collagen fibril organization in the enrichment analysis performed using GSVA (Figure S5). Several genes that encode collagen components were also upregulated in cluster 5 (Figure 2G). Considering the possible VSMC origin of this cluster of cells, we drew featureplots of the SMC marker genes *MYH11* and *CNN1* (Figure 2G). The featureplot showed that *MYH11* and *CNN1* exhibited low expression in all 5 clusters, indicating that cluster 5 is not derived from SMCs.

We next sought to explore how the transcriptional state of myeloid cells was regulated by TFs using single-cell regulatory network inference and clustering (SCENIC) analysis. We found that members of the nuclear factor-κB (NF-κB) TFs family were most significantly upregulated in cluster 2, which has been reported to be a key TF of M1 macrophages and to promote inflammatory gene expression (Figure 2H) (27, 28). X-box binding protein 1 (XBP1) was also reported to be important in regulating the expression of proinflammatory cytokines in activated macrophages (29). Several other TFs that were upregulated in cluster 2, including *CEBPB, FOSL2*, and *CREM*, were found to function in the process of monocyte differentiation into macrophages (30). In summary, the SCENIC analysis again indicated that the cluster 2 myeloid cells originated from circulatory monocytes. However, it also suggested that this cluster of myeloid cells displayed a predominantly proinflammatory phenotype.

### T and NK Cells Derived From AD Tissues Have Enhanced Cytotoxic Properties

In the current study, 6,168 NK and T cells were detected. These cells were further divided into 18 clusters using unsupervised clustering (Figures S6A,B). Based on the expression of *NKG7, CD3D, CD4*, and *CD8A* (Figure S6C), we divided these cells into NK cells (*n* = 1,668), CD4^+^ T cells (*n* = 2,489), and CD8^+^ T cells (*n* = 2,011) (Figure S6D). The relative abundances of CD4^+^ and CD8^+^ T cells were similar. Unsupervised reclustering was then performed in each cell type.

**Figure 3:**
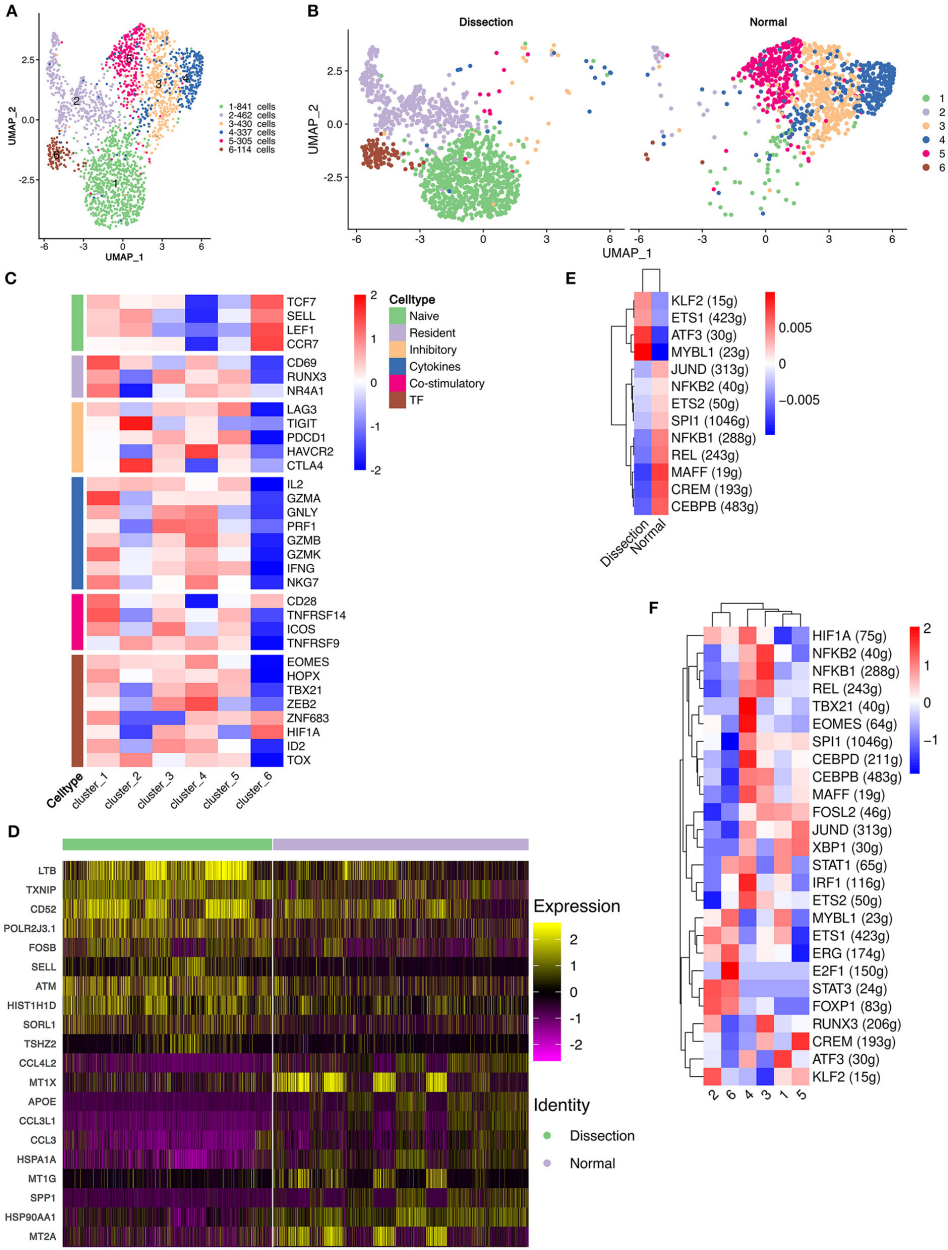
Functional changes in CD4^+^ T cells in AD tissues compared to normal aorta tissues. **(A)** Reclustering of CD4^+^ T cells into 6 clusters, as shown by the UMAP plot. **(B)** A UMAP plot showing CD4^+^ T cells from the AD and NA groups. **(C)** Heatmap of different properties of CD4^+^ T cells as identified by corresponding canonical markers. **(D)** Heatmap of the top 10 [by average log (fold change)] marker genes in CD4^+^ T cells in the AD and NA groups. **(E,F)** SCENIC analysis of the top differentially expressed transcription factors among different groups **(E)** or clusters of T cells **(F)**. AD, aortic dissection; NA, normal aorta; UMAP, uniform manifold approximation and projection for dimension reduction; SCENIC, single-cell regulatory network inference and clustering.

CD4^+^ T cells were reclustered into 6 clusters (Figure 3A). Significantly different distributions of CD4^+^ T cells were detected between AD and NA samples (Figure 3B). Clusters 1, 2, and 6 were primarily detected in AD samples, and clusters 3, 4, and 5 were primarily observed in NA samples.

Cluster 1 expressed *LTB*, which is important for the formation of the germinal center and promotes Th1-type immunity (Figure S7) (31, 32). However, well-defined marker genes for Th1 cells, e.g., *T-bet* and *IFN*γ (33), were not upregulated in cluster 1. Instead, cluster 1 expressed *CCR6*, which is a homing receptor for Th17 cells (33, 34). The costimulatory receptor-encoding genes *CD27, CD28* and integrin *ITGAL*, which mediate the migration of T cells into inflamed sites, were also expressed in cluster 1 (33). These results suggested that cluster 1 T cells were activated and were probably Th17 cells. Interestingly, markers for tissue resident memory T cells, including *CD69* and *CD103*, were also expressed in cluster 1 (33). In addition, cluster 1 expressed granzymes encoding the *GZMA, GZMK, GZMM* genes and the cytokine *IL32*, suggesting that this type of CD4^+^ T-cell might be involved in the direct promotion of apoptosis and the secretion of inflammatory factors. Cluster 2 was also expanded in the AD tissues. The top marker gene heatmap showed that cluster 2 was characterized by expressing genes encoding proteins of cellular components, including mitochondrial DNA *MT-ND3, MT-CO3, MT-ND1*, and *MT-CYB*, and ribosomal small subunit encoding genes *CMSS1* (Figure S7). These results indicated that the primary function of cluster 2 T cells was likely the biogenesis of organelles, especially mitochondria and ribosomes. Cluster 2 T cells also expressed the L-selectin-encoding gene *SELL*, which regulates T-cell homing to secondary lymphoid organs and is a marker for naïve and central memory T cells, while lacking other naïve T-cell markers, including *CCR7* and *IL7R* (Figure S7) (33). Therefore, these T cells were likely a cluster of naïve T cells experiencing phenotypic changes. Cluster 6 highly expressed the naïve T-cell markers *SELL, CCR7, LEF1*, and *TCF7* (Figure 3C and Figure S7). These T cells were defined as naïve T cells.

Cluster 3 T cells expressed apolipoprotein-encoding genes *APOE* and *APOC1* and cytokine-encoding genes *CCL3L1* and *CCL20*, indicating the lipid homeostasis maintenance function of this cluster of cells (Figure S7). Cluster 4 expressed heat shock protein-encoding genes, including *HSPA1A, HSPA1B, and HSPA6*, and cytokine-encoding genes *CCL4L2, CCL4*, and *CCL3* (Figure S7), suggesting that this cluster of T cells is involved in the stress response. Cluster 5 highly expressed metallothionein (MT)-encoding genes, including *MT1X, MT1G, MT1E, MT1F, and MT2A* (Figure S7). MTs can bind various heavy metals but primarily zinc in human tissue (35, 36). Under oxidative stress, zinc can be released from MTs, thereby contributing to suppression of oxidative stress (36).

We also compared the top DEGs of CD4^+^ T cells between the AD and NA groups (Figure 3D). In the AD group, consistent with the expansion of clusters 1, 2 and 6, the top DEGs included *LTB*, which was highly expressed in cluster 1. Genes that were usually highly expressed in naïve T cells, including *ATM* and *SELL* (37), were also upregulated in the AD group. In the NA group, these top DEGs were primarily those encoding MTs as well as genes related to lipid metabolism and stress response.

Next, using SCENIC analysis, we found that *ATF3* was one of the most highly expressed TFs in the AD group (Figure 3E). The *ATF3* gene was reported to promote human Th1 differentiation and *IL-17A* expression in Th17 cells (38, 39). The SCENIC analysis in different clusters showed that *ATF3* was primarily expressed in cluster 1 (Figure 3F). Overall, these results revealed that CD4^+^ T-cell clusters in the AD group had distinct functions from those in the NA group.

The cluster distribution of CD8^+^ T cells and NK cells was also different between the two groups (Figures S8A–D). The top DEGs of CD8^+^ T cells and NK cells between the two groups were similar to those of CD4^+^ T cells (Figures S8E,F). The CD8^+^ T cells and NK cells in the AD group exhibited elevated cytotoxic potential, while CD8^+^ T cells and NK cells in the NA group functioned primarily in lipid and zinc metabolism.

**Figure 4:**
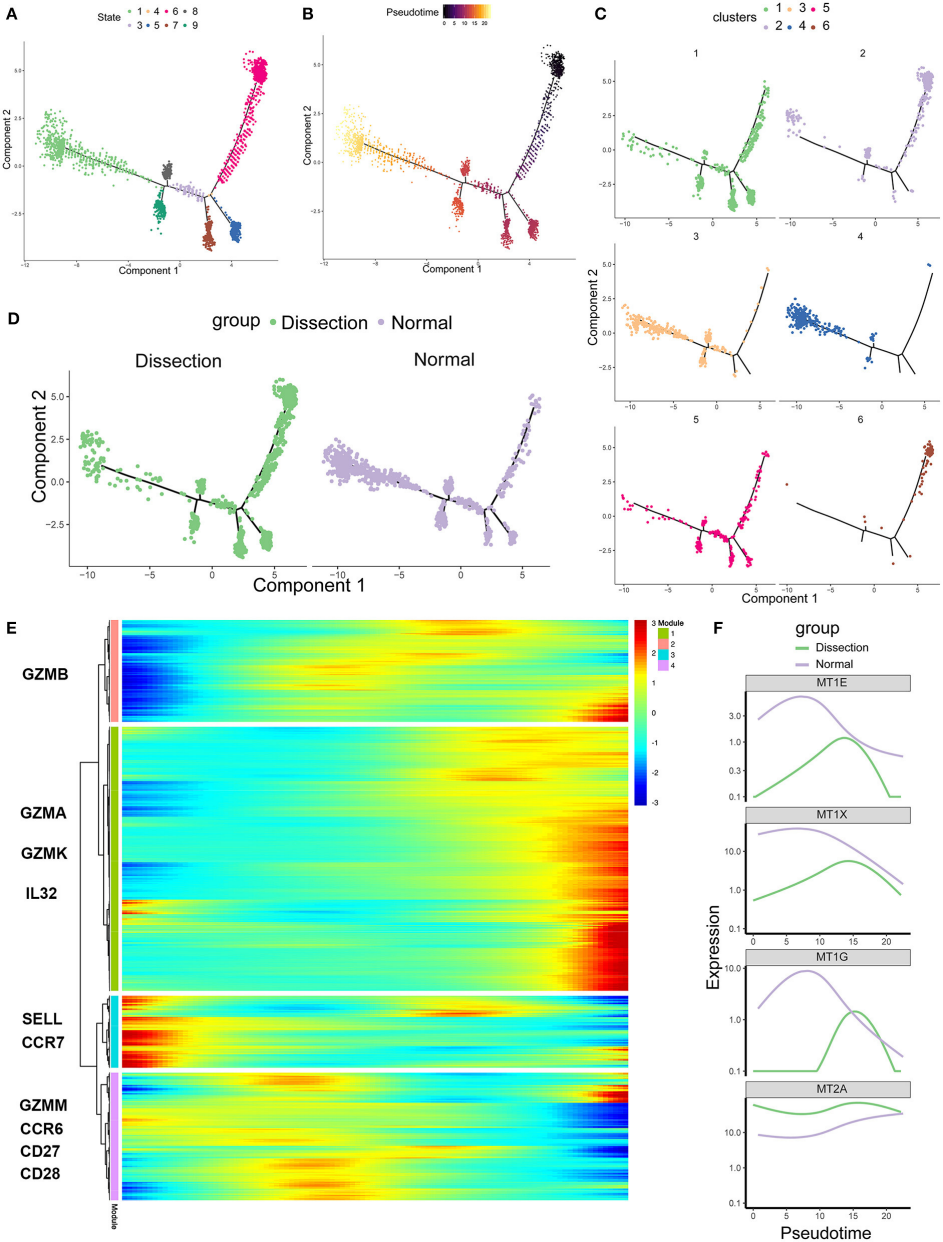
Single-cell trajectory analysis of CD4^+^ T cells based on Monocle. **(A)** The CD4^+^ T-cell trajectory was separated into 8 cell states. **(B)** State 6 was set as the starting point. **(C)** The CD4^+^ T-cell trajectories of different clusters. **(D)** The CD4^+^ T-cell trajectories of different groups. **(E)** Heatmap of genes that were significantly differentially expressed along the pseudotime axis. These genes were further clustered into 4 modules according to their expression patterns along the pseudotime axis. **(F)** Profiling of MTs along the trajectories of CD4^+^ T cells in the AD and NA groups. MTs, metallothioneins.

To further investigate the role of CD4^+^ T cells in AD, we applied pseudotime methods to simulate the differentiation trajectory of CD4^+^ T cells. Eight cell states were subsequently identified (Figure 4A). Based on the previous findings that cluster 6 primarily consisted of naïve CD4^+^ T cells, we used the branch of state 6 as the starting point (Figures 4B,C). Compared to the NA group, state 6 comprised more cells from the AD group (Figure 4D). This suggested that there were more naïve CD4^+^ T cells in the AD group, consistent with our previous finding that the two clusters expanded in the AD group, i.e., cluster 6 and cluster 2, were naïve CD4^+^ T cells and naïve CD4^+^ T cells experiencing phenotypic changes, respectively. Genes significantly differentially expressed along the pseudotime axis were then clustered into 4 modules according to their expression patterns in a heatmap (Figure 4E). Among them, modules 1 and 4 were genes whose expression levels first increased and then decreased along the pseudotime axis, module 2 was genes whose expression levels increased along the pseudotime axis, and module 3 was genes whose expression levels decreased along the pseudotime axis. Naïve CD4^+^ T-cell markers, such as *SELL* and *CCR7*, were identified in module 3. Several inflammatory proteins produced by cluster 1, including granzymes such as *GZMA* and *GZMK* and cytokines such as *IL32*, were identified in module 2. In addition, genes encoding MTs, including *MT1E, MT1G, MT1X, and MT2A*, were all identified in module 4 (Figure 4F). We examined the expression of MT-encoding genes along the pseudotime axis in the AD and NA groups (Figure 4F). These four MT-encoding genes all increase first and then decrease along the pseudotime axis, suggesting a transient increase in cellular zinc concentration after T-cell activation. However, expression level of MTs in the NA group was higher than that in the AD group on almost the entire pseudotime axis. This suggests that the increase in MTs in the NA group is more likely to be due to the higher zinc concentration in the normal aorta ECM rather than the recent activation of T cells (36, 40).

### B Cells as Antigen-Presenting Cells in the Dissected Aorta

**Figure 5:**
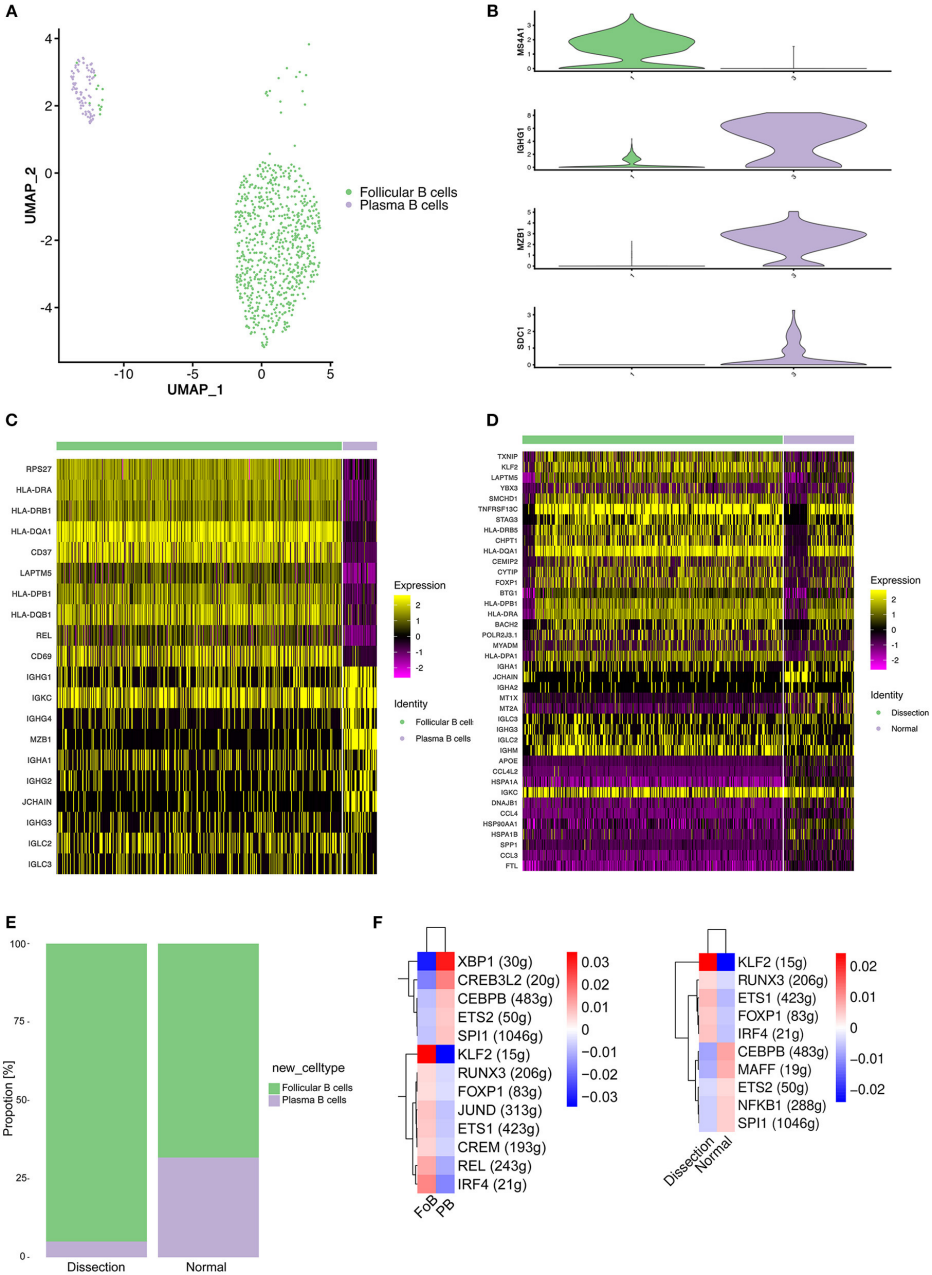
Profiling of B cells between normal and dissected aorta samples. **(A)** A UMAP plot of 832 B cells reclustered into 2 clusters. **(B)** UMAP plots showing the expression of selected marker genes for subpopulations of B cells. **(C)** Heatmap of the top 10 [by average log (fold change)] marker genes in FO B cells and plasma cells. **(D)** Heatmap of the top 20 [by average log (fold change)] marker genes in B cells of the AD and NA groups. **(E)** Bar graph showing the proportion of B-cell clusters in the AD and NA groups. **(F)** SCENIC analysis of the top differentially expressed transcription factors among different clusters (left) or groups of B cells (right). FO B cells, follicular B cells; UMAP, uniform manifold approximation and projection for dimension reduction; SCENIC, single-cell regulatory network inference and clustering; AD, aortic dissection; NA, normal aorta.

In our study, 832 B cells were detected, which were clustered into 2 groups (Figure 5A). Cluster 1 expressed the mature B-cell marker *MS4A1* (*CD20*) and was defined as follicular (FO) B cells. Cluster 2 exhibited marked upregulation of markers of plasma B cells, including *IGHG1, MZB1*, and *SDC1*, and was defined as plasma B cells (Figure 5B).

A heatmap displaying the expression of the top 10 most differentially expressed genes in each cluster (Figure 5C) revealed that FO B cells exhibited increased expression of MHC class II molecules (*HLA-DPB1, HLA-DQA1*, and *HLA-DQB1*), indicating that the primary function of this cluster of B cells might be antigen presentation.

We next compared the gene expression profile of B cells in the AD group to that in the NA group (Figure 5D). Significantly higher expression of MHC class II molecules was detected in the AD group. The B cells expressed MHC class II molecules and presented T-cell–dependent antigens to T cells, promoting the activation of both B and T cells. However, genes encoding proteins of immunoglobulin were upregulated in normal aorta, and *LAPTM5*, which negatively regulates B-cell activation (41), was highly expressed in the AD group, suggesting that the major role of B cells in AD was antigen presentation and activation of T cells. Consistently, the proportion of plasma B cells was higher in the NA group (Figure 5E).

We next sought to explore how the transcriptional state of B cells was regulated by TFs using SCENIC analysis (Figure 5F). Comparison between FO B cells and plasma cells revealed significantly upregulated expression of the TFs *KLF2, IRF4*, and *REL* (Figure 5F, left). The *KLF2* gene was found to be associated with the egress of FO B cells from the spleen and regulates the expression of the adhesion molecule *SELL*, which is critical for lymphocytes to home to peripheral lymph nodes (42). *IRF4* and *REL* were reported to be important for the proliferation of B cells (43). We further analyzed differences between the AD and NA groups (Figure 5F, right). A significant difference in the expression of *KLF2* still existed, while the expression of *IRF4* and *REL* in the AD group was less elevated than that in the NA group, suggesting that the B cells in the AD tissue might be primarily derived from the peripheral circulation.

### Increased Cell–Cell Communication Between NK/T Cells and Macrophages in AD Tissues

To further investigate the mutual regulatory process between the innate and acquired immune systems in AD tissues, we performed cell–cell communication analysis by mapping the ligand–receptor interactions with our scRNA-seq data.

**Figure 6:**
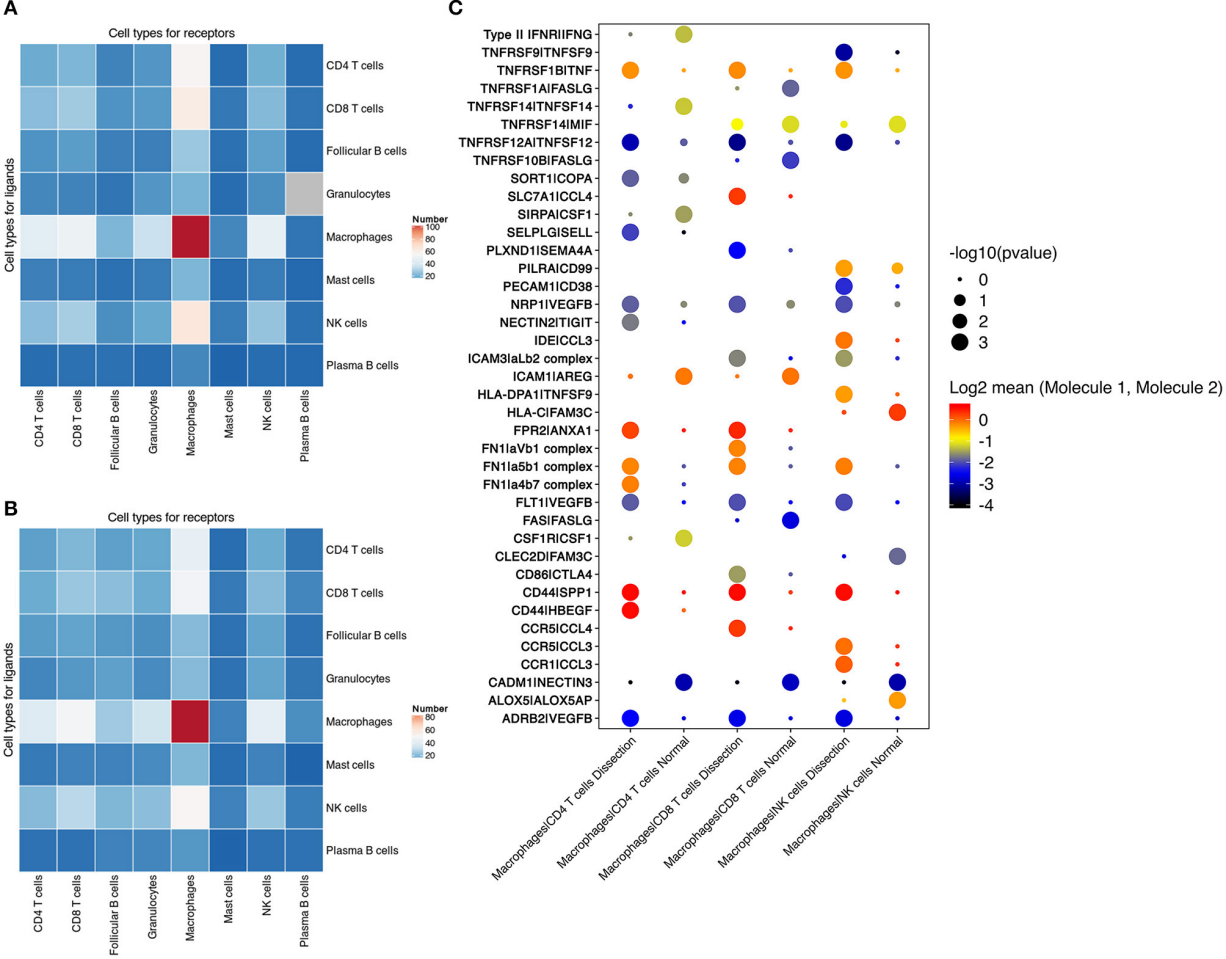
Cell–cell communication between immune cells. **(A,B)** Number of predicted interactions (*P* < 0.05) between macrophages, T cells, NK cells, B cells, neutrophils and mast cells based on CellPhoneDB in AD tissues **(A)** and normal aorta tissues **(B)**. **(C)** Selected ligand–receptor interactions between CD4^+^ T cells, CD8^+^ T cells, NK cells and macrophages, comparing the AD vs. NA group. AD, aortic dissection; NA, normal aorta.

We found that the largest number of ligand–receptor interactions occurred between macrophages and macrophages, indicating that both paracrine and autocrine signaling play a significant role in regulating macrophage functions (Figures 6A,B and Figure S9). In addition, compared to normal aorta, the signaling from CD4^+^ T cells, CD8^+^ T cells, NK cells to macrophages in the AD tissue was increased.

Cell–cell communication involving T and NK-cell costimulation was found to be upregulated in AD tissues. Elevation in the binding of the integrin α*L*β*2* complex (*LFA1*) to *ICAM3* was identified between CD8^+^ T cells, NK cells and macrophages. The binding of *LFA1* to *ICAM3* augments *LFA-1/ICAM-1*-mediated T-cell adhesion and functions in T-cell costimulation (44). The communication between integrins in CD4^+^ T cells, CD8^+^ T cells, NK cells, and fibronectin secreted by macrophages was also upregulated in AD tissues (Figure 6C).

T cells and NK cells also secrete cytokines to regulate macrophage functions. In AD tissues, elevated secretion of both *TNF* and *VEGF*β was found in NK and T cells, with increased expression of their corresponding receptors in macrophages. These results suggested that T cells in the AD tissue induced the differentiation of macrophages to both the M1 and M2 phenotypes.

### Validation

**Figure 7:**
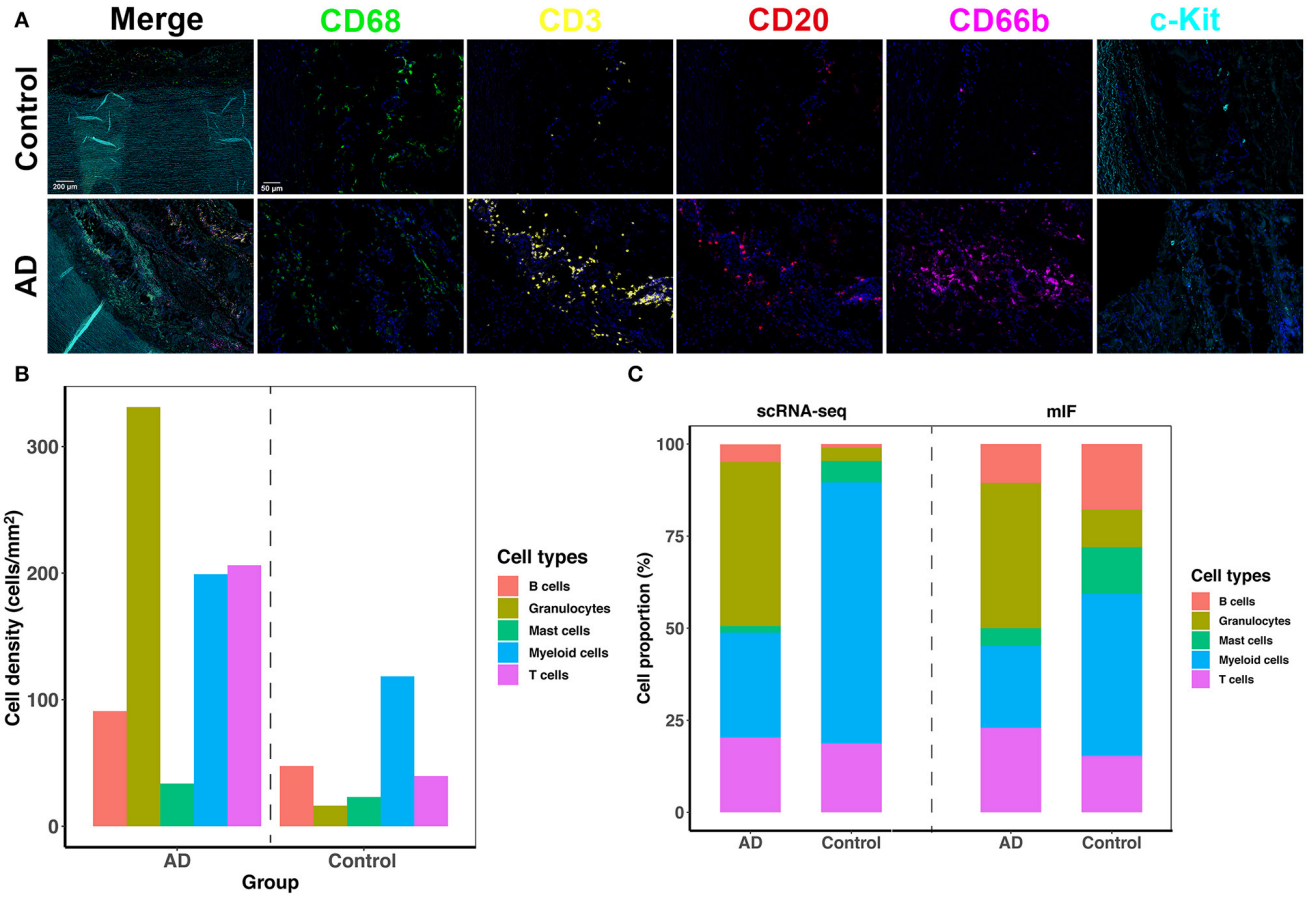
The five-plex mIF assay was performed in control and dissected aortas to validate the proportion of immune cells. **(A)** Representative mIF images of normal and dissected human ascending aortas stained with CD68 (green), CD3 (yellow), CD20 (red), CD66b (magenta), and c-Kit (cyan). **(B)** Bar graphs showing increased cell density of all five immune cell types in the AD tissue. **(C)** Bar graphs showing the percentage of immune cell populations from normal and dissected aortas identified by scRNA-seq analysis (2 bars on the left) or with mIF analysis (2 bars on the right). mIF: multiplexed immunofluorescence.

We adopted mIF to validate the overall proportion of each immune cell subtype (mast cells, granulocytes, B cells, macrophages, and T cells) present in control and dissected aorta tissues. All 5 immune cell subsets were successfully identified in both control and dissected aorta samples (Figure 7).

By comparing the density of immune cells between the two groups, we found that all immune cell subsets were expanded in AD tissues, and the expansion of granulocytes, T cells and myeloid cells was the most obvious (Figure 7B). The proportions of each immune cell subset identified using mIF vs. scRNA-seq were similar in AD tissues (Figure 7C). However, the findings of the proportion of immune cell subsets in normal aortas were not consistent between the two methods. This could be due to the limitation of mIF as a semiquantitative method, as well as individual differences in patients. In addition, since the total number of immune cells in normal aortic tissue was low, small deviations in cell counts could cause large changes in their percentage calculations. However, inconsistencies in these cell proportions should have little effect on the qualitative features of each cell subset.

## Discussion

### General Findings

Using scRNA-seq, we analyzed the immune cell subpopulations in sporadic human thoracic aortic dissection tissues. We found that after the onset of AD, 5 major types of immune cells expanded in AD tissue, among which granulocytes were predominant. The function of immune cells in AD tissues also changed, which manifested as a change in the distribution of cell subsets and different transcriptome profiles identified using scRNA-seq. Among them, macrophages and T cells exhibited the most prominent changes. Macrophages in normal aortas primarily function in lipid metabolism, whereas macrophages expanded in AD tissues primarily play a proinflammatory role. The functional changes of T cells in AD tissue were similar to those of macrophages. T cells were also primarily related to lipid metabolism and stress response in normal aorta, while the main function of the expanded T-cell subsets in AD tissues was to secrete inflammatory factors and granzymes. These expanded macrophages and T-cell subsets in AD tissues might contribute to the immune mechanism of AD.

### Infiltration of Circulating Macrophages Into the Aorta Might Contribute to Triggering Aortic Dissection

Activated macrophages can usually be simply divided into two major phenotypes, i.e., the proinflammatory M1 type and the anti-inflammatory M2 type. In studies concerning the immune mechanisms of aortic dissection, macrophages have usually been reported to manifest as the M1 phenotype and contribute to the occurrence of AD by secreting proteases and chemokines, which result in extracellular matrix degradation, elastic lamina breakdown and promotion of inflammatory responses (4).

In our study, the primary cluster expanded in AD tissues (cluster 2) was also predominantly a proinflammatory phenotype. However, unlike the classic M1-type macrophages reported to promote AD or aneurysmal diseases (45, 46), MMP-related genes were not found to be significantly elevated in cluster 2. In contrast, genes involved in ECM repair rather than degradation, especially *VCAN, EREG, AREG*, and *TIMP1*, were significantly expressed in cluster 2. This raises the question of whether the expansion of this cluster of macrophages is a consequence or a cause of AD. Although the findings of this study alone cannot answer this question, previous research might help shed light on this query. In the scRNA-seq study of ascending thoracic aortic aneurysm by Li et al. (6), a group of macrophages seemed to have a transcriptome similar to that of cluster 2 in our study, both of which significantly expressed inflammatory factors and tissue repair cytokines, including *TIMP1, VCAN, EREG*, and *AREG*. The chronic nature of aortic aneurysm development suggests that this population of macrophages is already present in the aortic wall, even in the absence of the event of AD onset, which suggests that the expansion of cluster 2 in the AD tissue is not simply the consequence of AD onset. In addition, we previously used BAPN and angiotensin II (Ang II) to create AD models in wild-type C57BL/6J mice and LysM^iDTR^ mice (47), in which monocytes in the peripheral blood were depleted after diphtheria toxin injection. The depletion of circulating monocytes significantly reduced the incidence of AD, indicating that the infiltration of macrophages in the aorta is more of a causal event of AD than simply a consequence of it. Notably, in this mouse AD model created using BAPN and Ang II, we only observed macrophage infiltration after dissection onset. This suggests that, unlike mouse AAA models, in which long-term infiltration of macrophages and chronic degradation of extracellular matrix can be observed (48, 49), macrophage infiltration in the AD model might act as a “trigger” event. Based on the medial VSMCs or ECM lesions, macrophages might infiltrate into the media and participate in ECM repair (46). However, proinflammatory cytokines can be produced in this process, which leads to chemotaxis and the activation of other immune cells, especially neutrophils and T cells. These immune cells could further secrete inflammatory factors and granzymes, aggravating the medial lesions and causing abrupt onset of aortic dissection.

### T Cells Might Play an Important Role in the Progression of AD

In our study, we found that T cells might play an important role in the development of AD. Similar to the study of Li et al. (6), we could not identify typical T-cell subtypes using their classical marker genes. However, a cluster of Th17-like T cells was found to expand in the AD group. Previous studies also reported elevation of Th17 cells both in AD tissues and blood samples of AD patients (50, 51). Ju et al. (52) observed Th17-cell accumulation in both patients and mouse models of aortic dissection. They found that Th17 cells contributed to the formation of AD primarily through the recruitment of macrophages. In our study, the cluster of Th17-like CD4^+^ T cells primarily secreted IL32, which induces macrophages to secrete inflammatory cytokines, including TNF-α (53). The recruitment of macrophages and secretion of TNF-α could further aggravate inflammatory responses and ECM degradation, thereby exacerbating the onset of AD. Moreover, these T cells also expressed the cytolytic molecules *GZMA, GZMK*, and *GZMM*, suggesting cytotoxic potential. Other cytotoxic immune cells, including CD8^+^ T cells and NK cells, also expressed higher levels of *GZMA, GZMK*, and *GZMH* in AD tissues. Deduction of the aortic wall cytotoxic T-cell content has been demonstrated to be one of the mechanisms of the inhibition of aneurysm formation by doxycycline (54).

Another interesting finding of this study was that the expression of MTs was upregulated in T cells of the normal aorta compared to the AD group. Combined with the pseudotime analysis, we found that this increase in MT expression might represent a higher zinc concentration in the normal aorta than in the dissected aorta. Edvinsson et al. found that Zn levels were decreased in human thoracic aortic dissection tissues and in the plasma of AD patients (55). Similar results were also detected in patients with AAA and TAA, suggesting that Zn levels in the plasma and aorta might influence the progression of aortic dissection and aneurysms (56, 57). Zinc deficiency has been reported to cause increased oxidative stress and autoreactive inflammation, which contribute to many cardiovascular diseases (36). Therefore, the upregulated MTs in our study may indicate a protective role of Zn in the progression of AD. However, the lower concentration of Zn in AD tissue could also be caused by higher expression of MMPs, which are Zn dependent. Further studies are required to determine the specific role of zinc in AD.

In the cell–cell communication study, we found that compared to the normal aorta, cellular connections were primarily increased between macrophages and T/NK cells. Most of these upregulated connections were related to T-cell activation, and T cells secrete TNF and VEGFβ to modulate the functions of macrophages. Together, these findings indicate that T cells might play an important role in the pathogenesis of AD by directly inducing cell death in AD tissues, as well as recruiting macrophages in turn and modulating their functions.

### Limitations

The sample size included in this study is relatively small; however, we enrolled a high number of immune cells for analysis after MACS sorting of CD45^+^ cells. Considering that heart transplants are infrequent and the high cost of scRNA-seq, we chose to load more cells from normal aorta onto the Chromium Single Cell Controller Instrument to obtain sufficient immune cells for the control group, which led to an abnormal increase in the proportion of immune cells in normal aortic tissue in our study. Another limitation of this study is that a large number of immune cells infiltrate into the aorta after AD onset, making it difficult to determine to what extent the described profiles are a consequence of dissection or its cause. Combined with previous studies, we preliminarily speculated that the macrophages expanded in the AD tissue in this study were likely to be the cause of the AD. More research is needed in the future to further clarify the role of these immune cells in the pathogenesis of AD. Finally, cluster 5 myeloid cells have also been found to function in connective tissue development and collagen fibril organization. It is therefore important to determine whether this cell cluster originates from VSMCs. Although the featureplot of *MYH11* and *CNN1* showed low expression of these two SMC marker genes in cluster 5, further confirmation with a higher level of evidence using reporter mice or *MYH11 in situ* Hybridization and Proximity Ligation Assays is required in the future (58).

## Conclusion

Herein, we revealed the landscape of immune cells in human thoracic aortic dissection tissues at the single-cell level. We identified a macrophage cluster with a unique transcriptome that was expanded in AD tissues. These macrophages might contribute to AD through secretion of cytokines and recruitment of other immune cells, especially granulocytes and T cells. Further investigation of the different roles of macrophages and T cells in AD may offer new diagnostic or treatment targets for AD.

## Data Availability Statement

According to national legislation/guidelines, specifically the Administrative Regulations of the People's Republic of China on Human Genetic Resources (http://www.gov.cn/zhengce/content/2019-06/10/content_5398829.htm, http://english.www.gov.cn/policies/latest_releases/2019/06/10/content_281476708945462.htm), no additional raw data is available at this time. Data of this project can be accessed after an approval application to the China National Genebank (CNGB, https://db.cngb.org/cnsa/). Please refer to https://db.cngb.org/, or email: CNGBdb@cngb.org for detailed application guidance. The accession code CNP0002621 should be included in the application.

## Ethics Statement

The studies involving human participants were reviewed and approved by Ethical Committee of Zhongshan Hospital, Fudan University. The patients/participants provided their written informed consent to participate in this study.

## Author Contributions

YL, LZ, WF, ZD, and BJ: conception and design. YL, LZ, HL, and XJ: sample collection. YL, JL, HL, and XJ: preparation of single cell suspensions and MACS sorting. YL, LZ, HT, and JL: single cell RNA sequencing and single-cell RNA-seq data processing. YL, LZ, and HT: writing the manuscript. WF, ZD, and BJ: critical revision and overall responsibility. YL and JL: statistical analysis. WF and ZD: obtained funding. All authors have reviewed the manuscript and approved the final version.

## Funding

This work was sponsored by National Natural Science Foundation of China (Nos. 81970395 and 81770508), the Project of Outstanding Academic Leaders of Shanghai Science and Technology Commission (No. 19XD1401200), and Shanghai Interventional Therapy Engineering Technology Research Center (No. 19DZ2250300).

## Conflict of Interest

The authors declare that the research was conducted in the absence of any commercial or financial relationships that could be construed as a potential conflict of interest.

## Publisher's Note

All claims expressed in this article are solely those of the authors and do not necessarily represent those of their affiliated organizations, or those of the publisher, the editors and the reviewers. Any product that may be evaluated in this article, or claim that may be made by its manufacturer, is not guaranteed or endorsed by the publisher.

## References

[1] Nienaber CA Clough RE Sakalihasan N Suzuki T Gibbs R Mussa F. Aortic dissection. Nat Rev Dis Primers. (2016) 2:16053. 10.1038/nrdp.2016.53 27440162

[2] Shen YH LeMaire SA Webb NR Cassis LA Daugherty A Lu S. Aortic aneurysms and dissections series. Arterioscler Thromb Vasc Biol. (2020) 40:e37–46. 10.1161/ATVBAHA.120.313804 32101472 PMC7233726

[3] Cifani N Proietta M Tritapepe L Di Gioia C Ferri L Taurino M. Stanford-A acute aortic dissection, inflammation, and metalloproteinases: a review. Ann Med. (2015) 47:441–6. 10.3109/07853890.2015.1073346 26339779

[4] Khoury MK Yang H Liu B. Macrophage biology in cardiovascular diseases. Arterioscler Thromb Vasc Biol. (2021) 41:e77–81. 10.1161/ATVBAHA.120.313584 33054391 PMC8046835

[5] Papalexi E Satija R. Single-cell RNA sequencing to explore immune cell heterogeneity. Nat Rev Immunol. (2018) 18:35–45. 10.1038/nri.2017.76 28787399

[6] Li Y Ren P Dawson A Vasquez HG Ageedi W Zhang C. Single-cell transcriptome analysis reveals dynamic cell populations and differential gene expression patterns in control and aneurysmal human aortic tissue. Circulation. (2020) 142:1374–88. 10.1161/CIRCULATIONAHA.120.046528 33017217 PMC7539140

[7] Pedroza AJ Tashima Y Shad R Cheng P Wirka R Churovich S. Single-cell transcriptomic profiling of vascular smooth muscle cell phenotype modulation in Marfan syndrome aortic aneurysm. Arterioscler Thromb Vasc Biol. (2020) 40:2195–211. 10.1161/ATVBAHA.120.314670 32698686 PMC7484233

[8] Yang H Zhou T Stranz A DeRoo E Liu B. Single-cell RNA sequencing reveals heterogeneity of vascular cells in early stage murine abdominal aortic aneurysm-brief report. Arterioscler Thromb Vasc Biol. (2021) 41:1158–66. 10.1161/ATVBAHA.120.315607 33472403 PMC7904588

[9] Zhao G Lu H Chang Z Zhao Y Zhu T Chang L. Single-cell RNA sequencing reveals the cellular heterogeneity of aneurysmal infrarenal abdominal aorta. Cardiovasc Res. (2021) 117:1402–16. 10.1093/cvr/cvaa214 32678909 PMC8064434

[10] Cochain C Vafadarnejad E Arampatzi P Pelisek J Winkels H Ley K. Single-Cell RNA-Seq reveals the transcriptional landscape and heterogeneity of aortic macrophages in murine atherosclerosis. Circ Res. (2018) 122:1661–74. 10.1161/CIRCRESAHA.117.312509 29545365

[11] Winkels H Ehinger E Vassallo M Buscher K Dinh HQ Kobiyama K. Atlas of the immune cell repertoire in mouse atherosclerosis defined by single-cell RNA-sequencing and mass cytometry. Circ Res. (2018) 122:1675–88. 10.1161/CIRCRESAHA.117.312513 29545366 PMC5993603

[12] Martini E Kunderfranco P Peano C Carullo P Cremonesi M Schorn T. Single-cell sequencing of mouse heart immune infiltrate in pressure overload-driven heart failure reveals extent of immune activation. Circulation. (2019) 140:2089–107. 10.1161/CIRCULATIONAHA.119.041694 31661975

[13] Butcher MJ Herre M Ley K Galkina E. Flow cytometry analysis of immune cells within murine aortas. J Vis Exp. (2011) 53, 2848. 10.3791/2848 PMC3196167 21750492

[14] Butler A Hoffman P Smibert P Papalexi E Satija R. Integrating single-cell transcriptomic data across different conditions, technologies, and species. Nat Biotechnol. (2018) 36:411–20. 10.1038/nbt.4096 29608179 PMC6700744

[15] Macosko EZ Basu A Satija R Nemesh J Shekhar K Goldman M. Highly parallel genome-wide expression profiling of individual cells using nanoliter droplets. Cell. (2015) 161:1202–14. 10.1016/j.cell.2015.05.002 26000488 PMC4481139

[16] Haghverdi L Lun ATL Morgan MD Marioni J.C. Batch effects in single-cell RNA-sequencing data are corrected by matching mutual nearest neighbors. Nat Biotechnol. (2018) 36:421–7. 10.1038/nbt.4091 29608177 PMC6152897

[17] Hänzelmann S Castelo R Guinney J. GSVA: gene set variation analysis for microarray and RNA-seq data. BMC Bioinformatics. (2013) 14:7. 10.1186/1471-2105-14-7 23323831 PMC3618321

[18] Trapnell C Cacchiarelli D Grimsby J Pokharel P Li S Morse M. The dynamics and regulators of cell fate decisions are revealed by pseudotemporal ordering of single cells. Nat Biotechnol. (2014) 32:381–6. 10.1038/nbt.2859 24658644 PMC4122333

[19] Aibar S González-Blas CB Moerman T Huynh-Thu VA Imrichova H Hulselmans G. SCENIC: single-cell regulatory network inference and clustering. Nat Methods. (2017) 14:1083–6. 10.1038/nmeth.4463 28991892 PMC5937676

[20] Efremova M Vento-Tormo M Teichmann SA Vento-Tormo R. CellPhoneDB: inferring cell-cell communication from combined expression of multi-subunit ligand-receptor complexes. Nat Protoc. (2020) 15:1484–506. 10.1038/s41596-020-0292-x 32103204

[21] Taube JM Roman K Engle EL Wang C Ballesteros-Merino C Jensen SM. Multi-institutional TSA-amplified Multiplexed Immunofluorescence Reproducibility Evaluation (MITRE) study. J Immunother Cancer. (2021) 9. 10.1136/jitc-2020-002197 PMC8286792 34266881

[22] Collin M Bigley V. Human dendritic cell subsets: an update. Immunology. (2018) 154:3–20. 10.1111/imm.12888 29313948 PMC5904714

[23] Aoki H Majima R Hashimoto Y Hirakata S Ohno-Urabe S. Ying and Yang of Stat3 in pathogenesis of aortic dissection. J Cardiol. (2021) 77:471–4. 10.1016/j.jjcc.2020.10.010 33148468

[24] Sun Y Wu L Zhong Y Zhou K Hou Y Wang Z. Single-cell landscape of the ecosystem in early-relapse hepatocellular carcinoma. Cell. (2021) 184:404–21.e416. 10.1016/j.cell.2020.11.041 33357445

[25] Oda T Hirota K Nishi K Takabuchi S Oda S Yamada H. Activation of hypoxia-inducible factor 1 during macrophage differentiation. Am J Physiol Cell Physiol. (2006) 291:C104–13. 10.1152/ajpcell.00614.2005 16481368

[26] Malyshev I Malyshev Y. Current concept and update of the macrophage plasticity concept: intracellular mechanisms of reprogramming and M3 macrophage “switch” phenotype. Biomed Res Int. (2015) 2015:341308. 10.1155/2015/341308 26366410 PMC4561113

[27] Lawrence T. The nuclear factor NF-kappaB pathway in inflammation. Cold Spring Harb Perspect Biol. (2009) 1:a001651. 10.1101/cshperspect.a001651 20457564 PMC2882124

[28] Dorrington MG Fraser IDC. NF-κB signaling in macrophages: dynamics, crosstalk, and signal integration. Front Immunol. (2019) 10:705. 10.3389/fimmu.2019.00705 31024544 PMC6465568

[29] He Y Sun S Sha H Liu Z Yang L Xue Z. Emerging roles for XBP1, a sUPeR transcription factor. Gene Expr. (2010) 15:13–25. 10.3727/105221610X12819686555051 21061914 PMC3374844

[30] Saeed S Quintin J Kerstens HH Rao NA Aghajanirefah A Matarese F. Epigenetic programming of monocyte-to-macrophage differentiation and trained innate immunity. Science. (2014) 345:1251086. 10.1126/science.1251086 25258085 PMC4242194

[31] Gräbner R Lötzer K Döpping S Hildner M Radke D Beer M. Lymphotoxin beta receptor signaling promotes tertiary lymphoid organogenesis in the aorta adventitia of aged ApoE-/- mice. J Exp Med. (2009) 206:233–48. 10.1084/jem.20080752 19139167 PMC2626665

[32] Upadhyay V Fu YX. Lymphotoxin signalling in immune homeostasis and the control of microorganisms. Nat Rev Immunol. (2013) 13:270–9. 10.1038/nri3406 23524463 PMC3900493

[33] Brummelman J Pilipow K Lugli E. The single-cell phenotypic identity of human CD8(+) and CD4(+) T cells. Int Rev Cell Mol Biol. (2018) 341:63–124. 10.1016/bs.ircmb.2018.05.007 30262035

[34] Wang C Kang SG Lee J Sun Z Kim C.H. The roles of CCR6 in migration of Th17 cells and regulation of effector T-cell balance in the gut. Mucosal Immunol. (2009) 2:173–83. 10.1038/mi.2008.84 19129757 PMC2709747

[35] Singer M Wang C Cong L Marjanovic ND Kowalczyk MS Zhang H. A distinct gene module for dysfunction uncoupled from activation in tumor-infiltrating T cells. Cell. (2017) 171:1221–3. 10.1016/j.cell.2017.11.006 29149608 PMC5788298

[36] Choi S Liu X Pan Z. Zinc deficiency and cellular oxidative stress: prognostic implications in cardiovascular diseases. Acta Pharmacol Sin. (2018) 39:1120–32. 10.1038/aps.2018.25 29926844 PMC6289396

[37] Zhao J Dang X Zhang P Nguyen LN Cao D Wang L. Insufficiency of DNA repair enzyme ATM promotes naive CD4 T-cell loss in chronic hepatitis C virus infection. Cell Discov. (2018) 4:16. 10.1038/s41421-018-0015-4 29644094 PMC5891503

[38] Filén S Ylikoski E Tripathi S West A Björkman M Nyström J. Activating transcription factor 3 is a positive regulator of human IFNG gene expression. J Immunol. (2010) 184:4990–9. 10.4049/jimmunol.0903106 20304822

[39] Escobar TM Kanellopoulou C Kugler DG Kilaru G Nguyen CK Nagarajan V. miR-155 activates cytokine gene expression in Th17 cells by regulating the DNA-binding protein Jarid2 to relieve polycomb-mediated repression. Immunity. (2014) 40:865–79. 10.1016/j.immuni.2014.03.014 24856900 PMC4092165

[40] Bonaventura P Benedetti G Albarède F Miossec P. Zinc and its role in immunity and inflammation. Autoimmun Rev. (2015) 14:277–85. 10.1016/j.autrev.2014.11.008 25462582

[41] Ouchida R Kurosaki T Wang JY. A role for lysosomal-associated protein transmembrane 5 in the negative regulation of surface B cell receptor levels and B cell activation. J Immunol. (2010) 185:294–301. 10.4049/jimmunol.1000371 20519653

[42] Winkelmann R Sandrock L Porstner M Roth E Mathews M Hobeika E. B cell homeostasis and plasma cell homing controlled by Krüppel-like factor 2. Proc Natl Acad Sci USA. (2011) 108:710–5. 10.1073/pnas.1012858108 21187409 PMC3021026

[43] Grumont RJ Gerondakis S. Rel induces interferon regulatory factor 4 (IRF-4) expression in lymphocytes: modulation of interferon-regulated gene expression by rel/nuclear factor kappaB. J Exp Med. (2000) 191:1281–92. 10.1084/jem.191.8.1281 10770796 PMC2193138

[44] Bleijs DA Binnerts ME van Vliet SJ Figdor CG van Kooyk Y. Low-affinity LFA-1/ICAM-3 interactions augment LFA-1/ICAM-1-mediated T cell adhesion and signaling by redistribution of LFA-1. J Cell Sci. (2000) 113 (Pt. 3):391–400. 10.1242/jcs.113.3.391 10639327

[45] Cheng Z Zhou YZ Wu Y Wu QY Liao XB Fu XM. Diverse roles of macrophage polarization in aortic aneurysm: destruction and repair. J Transl Med. (2018) 16:354. 10.1186/s12967-018-1731-0 30545380 PMC6293547

[46] Wang X Zhang H Cao L He Y Ma A Guo W. The role of macrophages in aortic dissection. Front Physiol. (2020) 11:54. 10.3389/fphys.2020.00054 32116765 PMC7013038

[47] Li X Liu D Zhao L Wang L Li Y Cho K. Targeted depletion of monocyte/macrophage suppresses aortic dissection with the spatial regulation of MMP-9 in the aorta. Life Sci. (2020) 254:116927. 10.1016/j.lfs.2019.116927 31672577

[48] Liu Z Morgan S Ren J Wang Q Annis DS Mosher DF. Thrombospondin-1 (TSP1) contributes to the development of vascular inflammation by regulating monocytic cell motility in mouse models of abdominal aortic aneurysm. Circ Res. (2015) 117:129–41. 10.1161/CIRCRESAHA.117.305262 25940549 PMC4490953

[49] Yang H Zhou T Sorenson CM Sheibani N Liu B. Myeloid-derived TSP1 (thrombospondin-1) contributes to abdominal aortic aneurysm through suppressing tissue inhibitor of metalloproteinases-1. Arterioscler Thromb Vasc Biol. (2020) 40:e350–66. 10.1161/ATVBAHA.120.314913 33028100 PMC7686278

[50] Ye J Wang Y Wang Z Ji Q Huang Y Zeng T. Circulating Th1, Th2, Th9, Th17, Th22, and Treg levels in aortic dissection patients. Mediat Inflamm. (2018) 2018:5697149. 10.1155/2018/5697149 30258282 PMC6146596

[51] Chen F Han J Tang B. Patterns of immune infiltration and the key immune-related genes in acute type A aortic dissection in bioinformatics analyses. Int J Gen Med. (2021) 14:2857–69. 10.2147/IJGM.S317405 34211294 PMC8242140

[52] Ju X Ijaz T Sun H Ray S Lejeune W Lee C. Interleukin-6-signal transducer and activator of transcription-3 signaling mediates aortic dissections induced by angiotensin II via the T-helper lymphocyte 17-interleukin 17 axis in C57BL/6 mice. Arterioscler Thromb Vasc Biol. (2013) 33:1612–21. 10.1161/ATVBAHA.112.301049 23685554 PMC3818154

[53] Khawar MB Abbasi MH Sheikh N. IL-32: a novel pluripotent inflammatory interleukin, towards gastric inflammation, gastric cancer, and chronic rhino sinusitis. Mediators Inflamm. (2016) 2016:8413768. 10.1155/2016/8413768 27143819 PMC4837279

[54] Lindeman JH Abdul-Hussien H van Bockel JH Wolterbeek R Kleemann R. Clinical trial of doxycycline for matrix metalloproteinase-9 inhibition in patients with an abdominal aneurysm: doxycycline selectively depletes aortic wall neutrophils and cytotoxic T cells. Circulation. (2009) 119:2209–16. 10.1161/CIRCULATIONAHA.108.806505 19364980

[55] Edvinsson M Ilbäck NG Frisk P Thelin S Nyström-Rosander C. Trace element changes in thoracic aortic dissection. Biol Trace Elem Res. (2016) 169:159–63. 10.1007/s12011-015-0432-2 26152852

[56] Chen T Zhang H Zhang Y Yang M Wu J Yang M. Association of circulating and aortic zinc and copper levels with clinical abdominal aortic aneurysm: a meta-analysis. Biol Trace Elem Res. (2021) 199:513–26. 10.1007/s12011-020-02187-8 32557106

[57] Socha K Karwowska A Kurianiuk A Markiewicz-Zukowska R Guzowski A Gacko M. Estimation of selected minerals in aortic aneurysms-impaired ratio of zinc to lead may predispose? Biol Trace Elem Res. (2021) 199:2811–18. 10.1007/s12011-020-02410-6 33006035 PMC8222018

[58] Gomez D Shankman LS Nguyen AT Owens G.K. Detection of histone modifications at specific gene loci in single cells in histological sections. Nat Methods. (2013) 10:171–7. 10.1038/nmeth.2332 23314172 PMC3560316

