# Supplementary figures and images for: Single-Cell Sequencing of Immune Cells in Human Aortic Dissection Tissue Provides Insights Into Immune Cell Heterogeneity
Source: Front Cardiovasc Med. 2022 Mar 31;9:791875. doi: 10.3389/fcvm.2022.791875 (PMC9008490; doi:10.3389/fcvm.2022.791875)

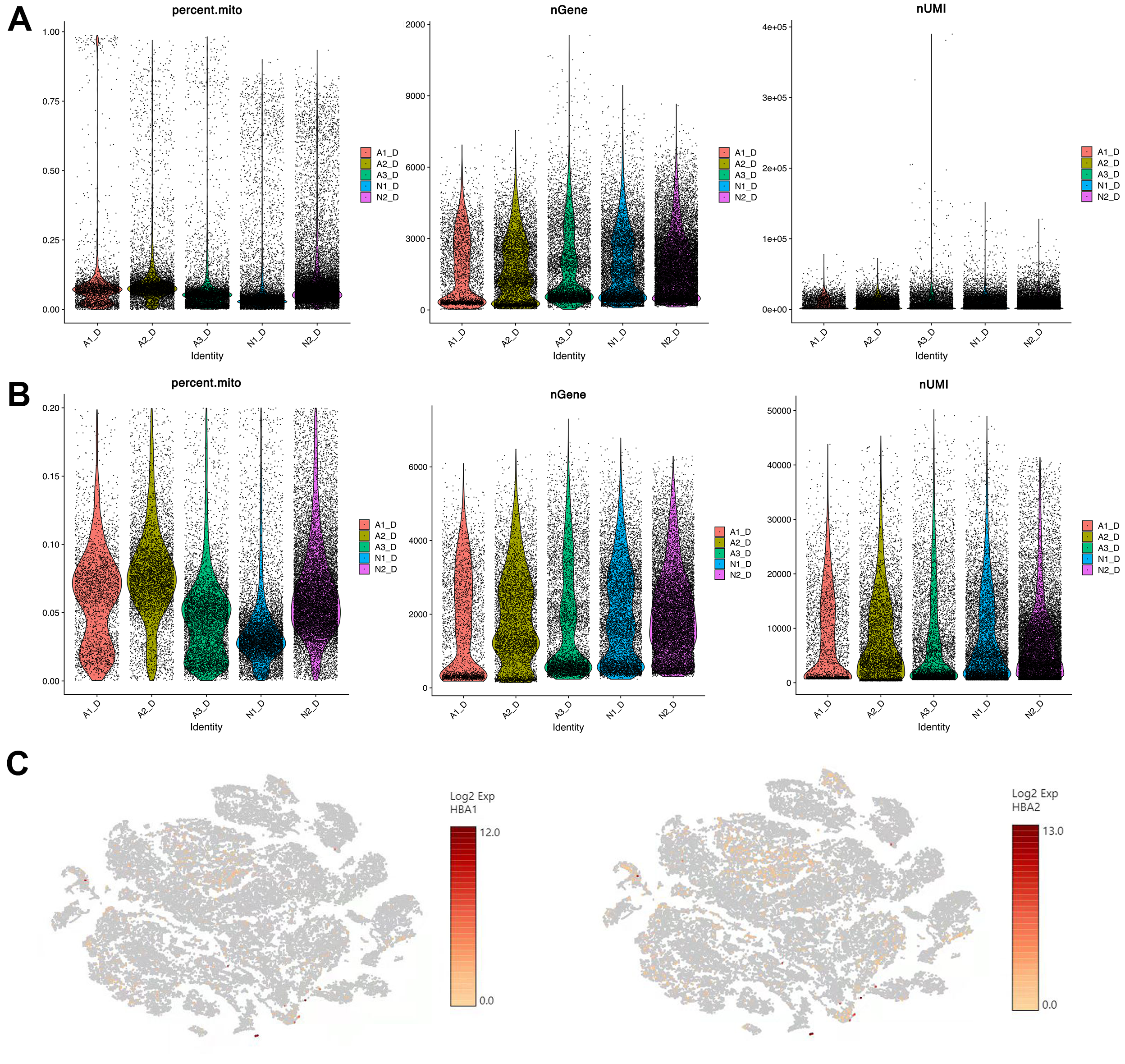

Supplement: Supplementary file 1 [file Image_1.TIF]

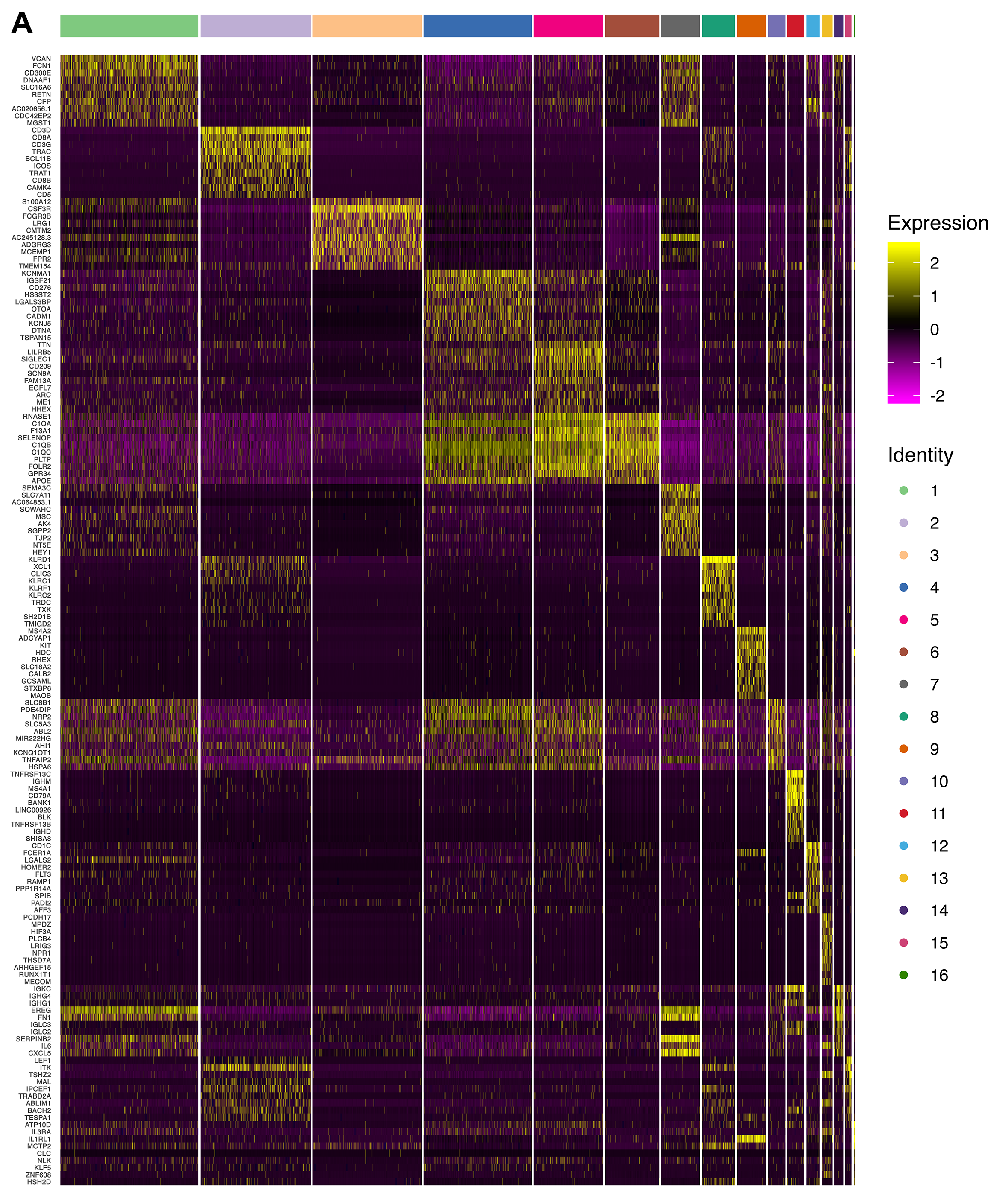

Supplement: Supplementary file 2 [file Image_2.TIF]

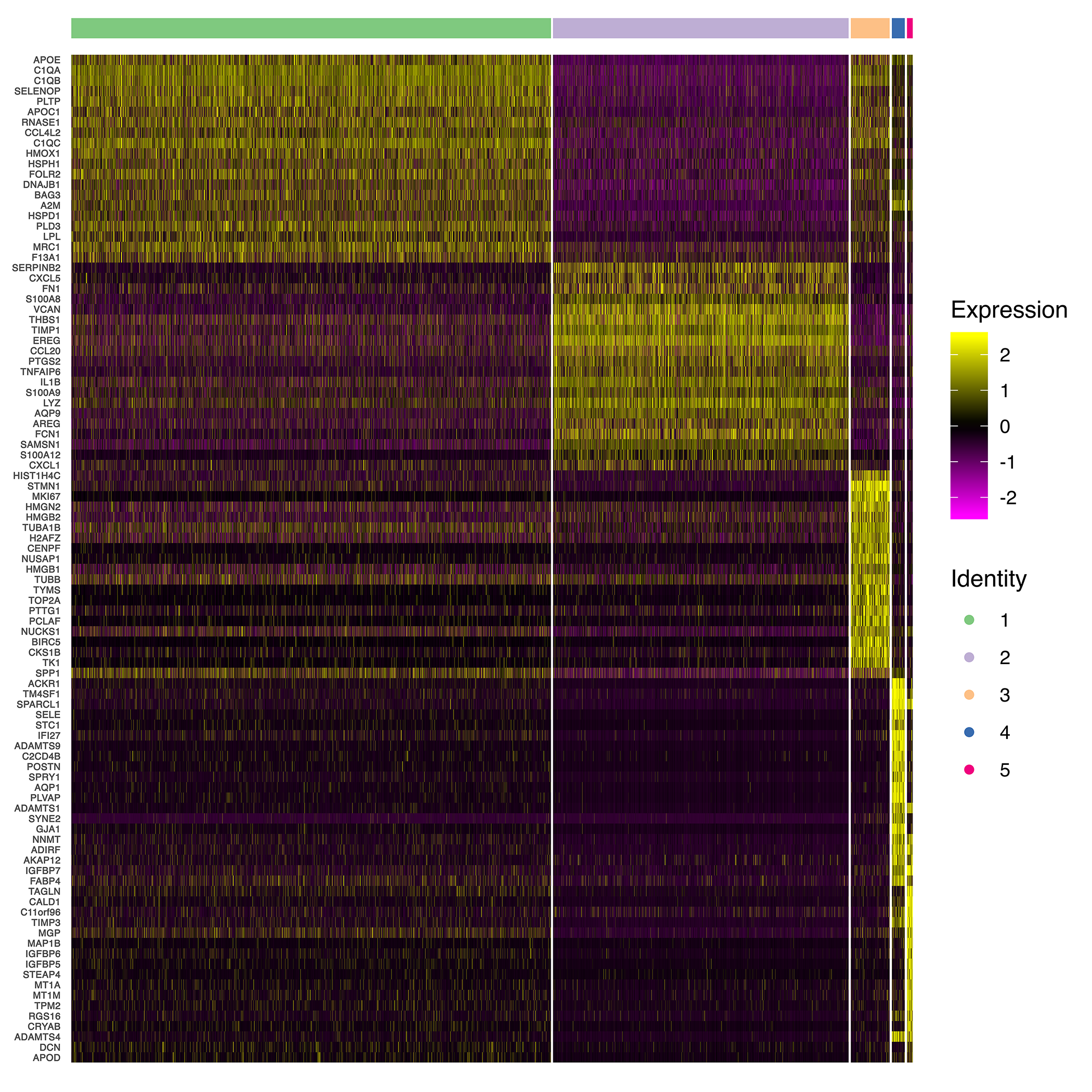

Supplement: Supplementary file 3 [file Image_3.TIF]

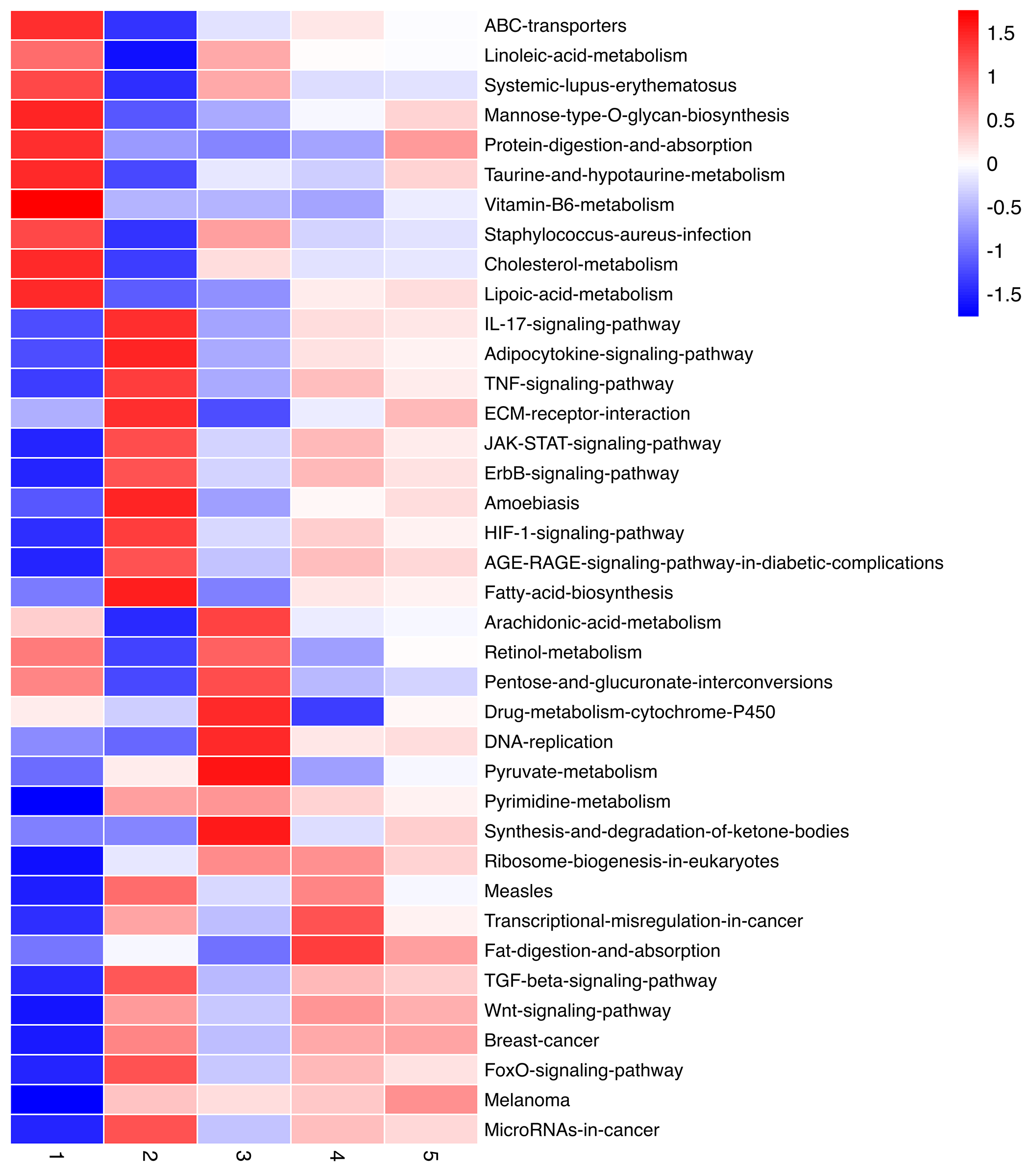

Supplement: Supplementary file 4 [file Image_4.TIF]

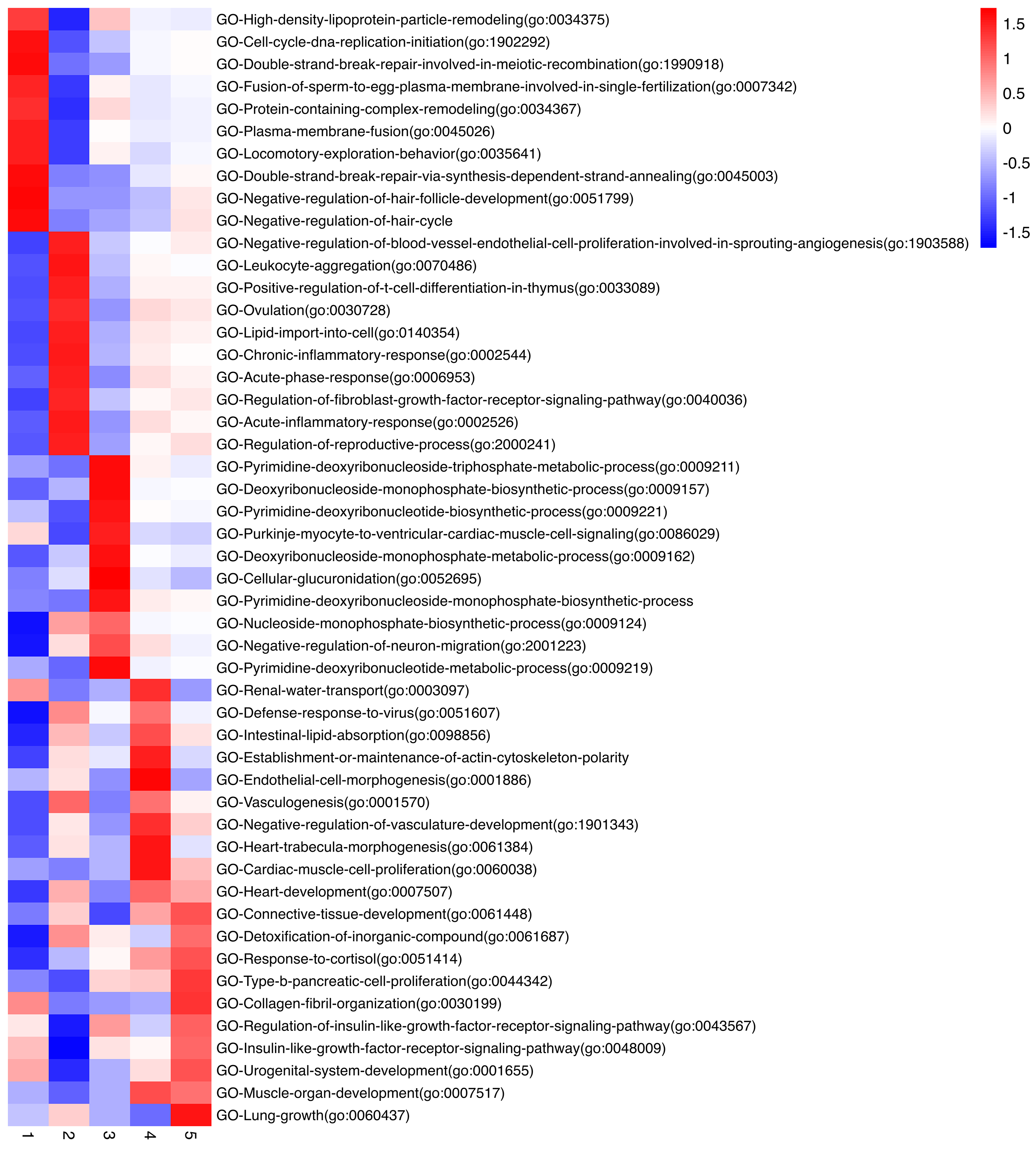

Supplement: Supplementary file 5 [file Image_5.TIF]

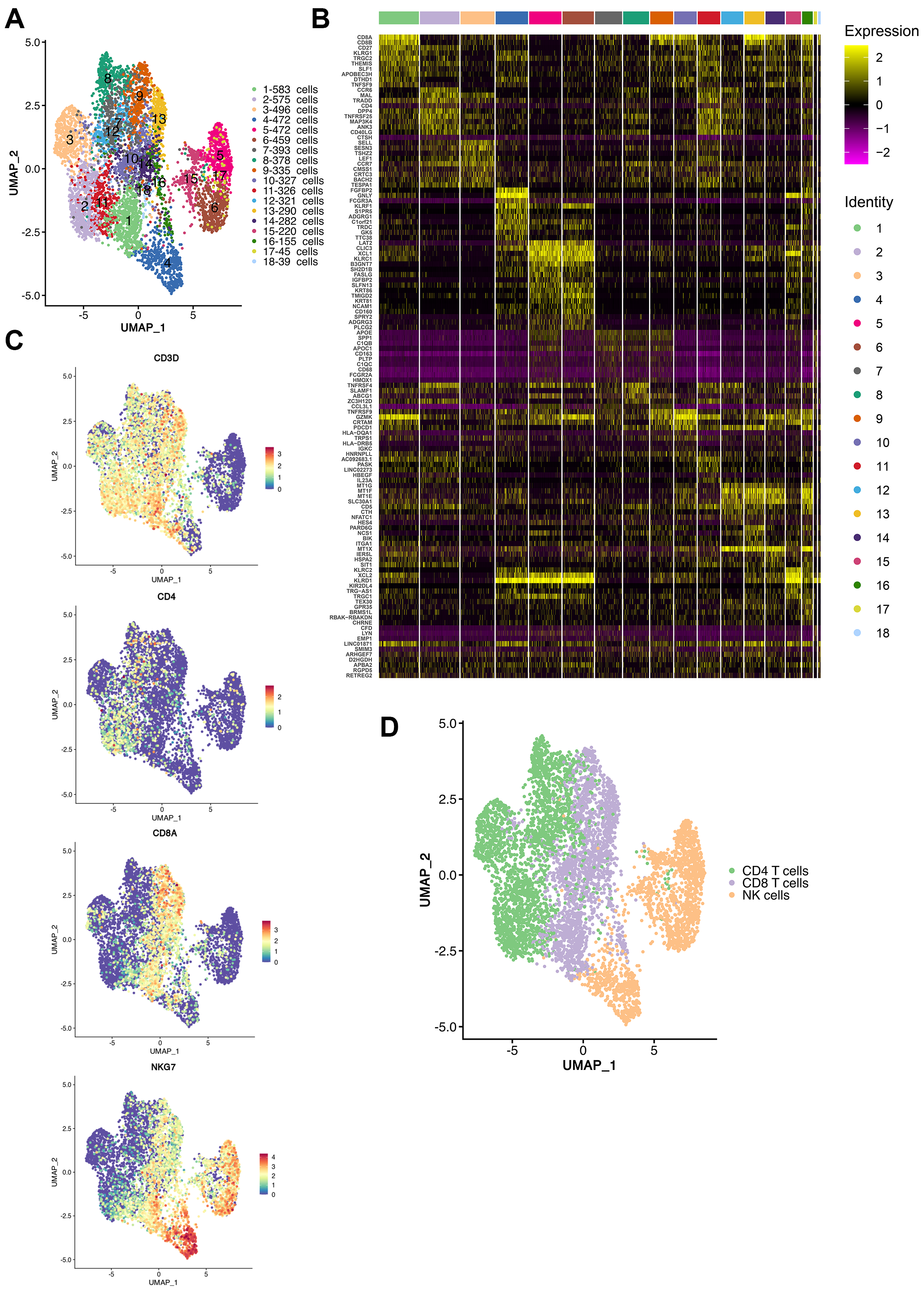

Supplement: Supplementary file 6 [file Image_6.TIF]

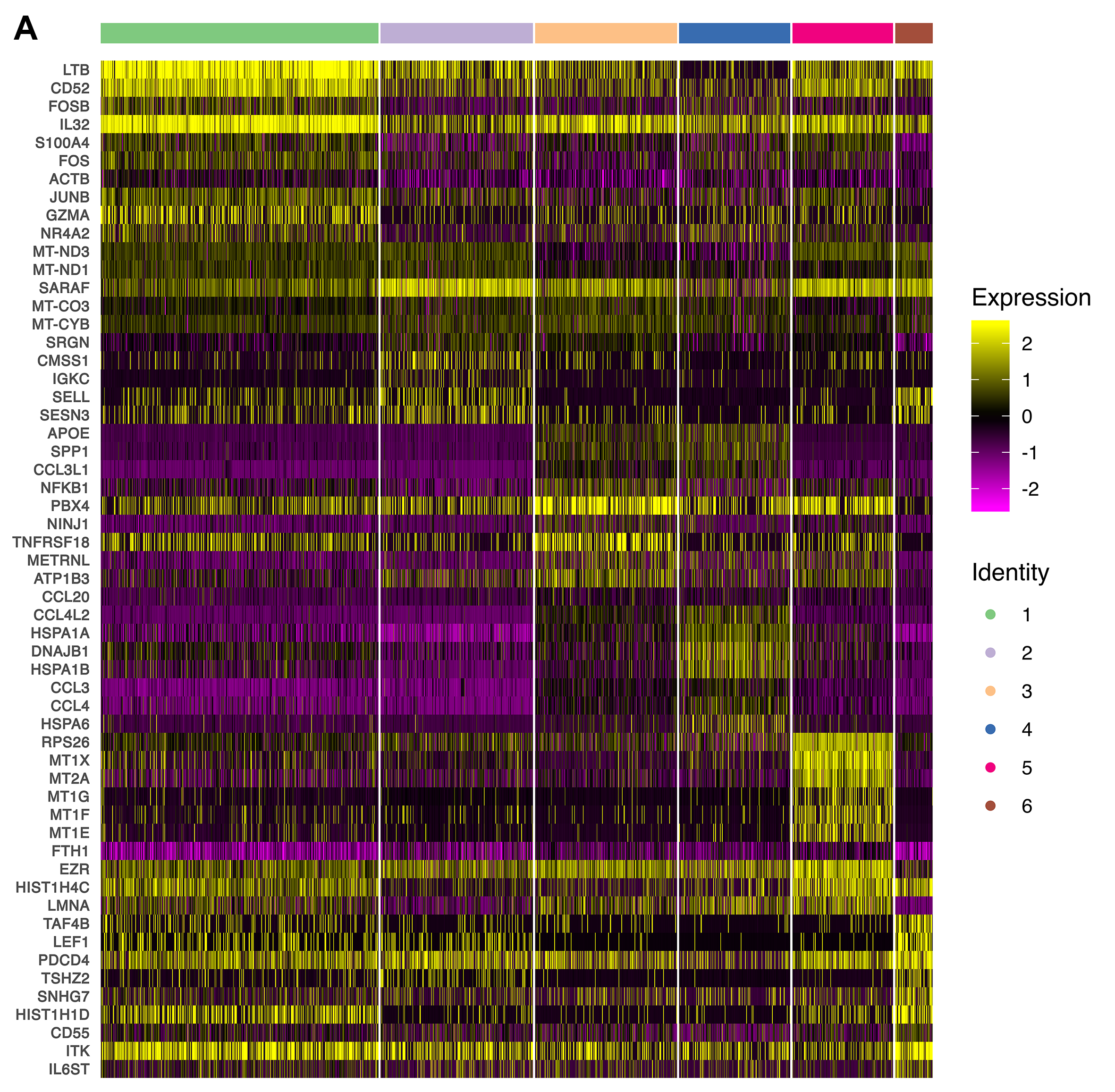

Supplement: Supplementary file 7 [file Image_7.TIF]

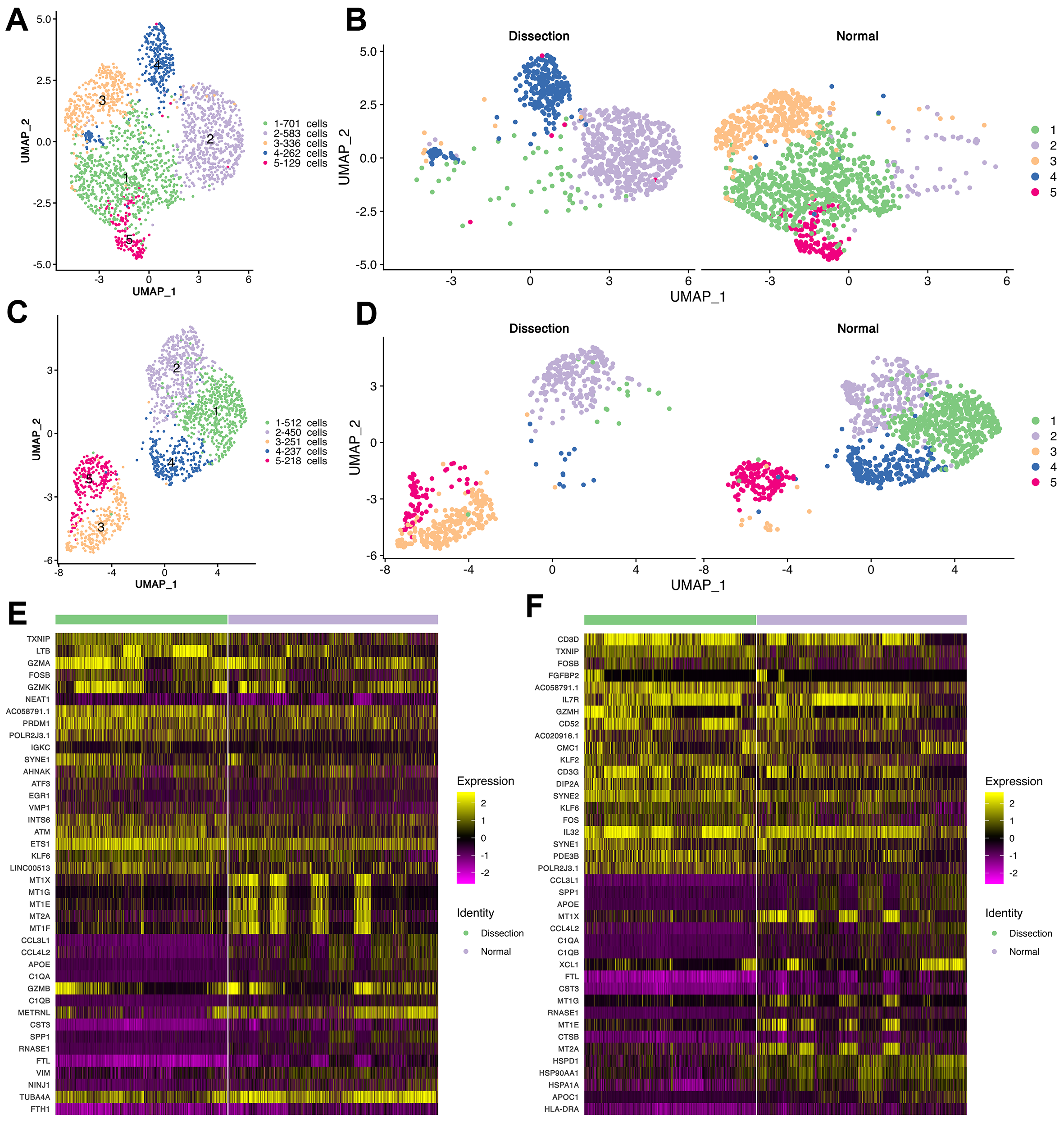

Supplement: Supplementary file 8 [file Image_8.TIF]

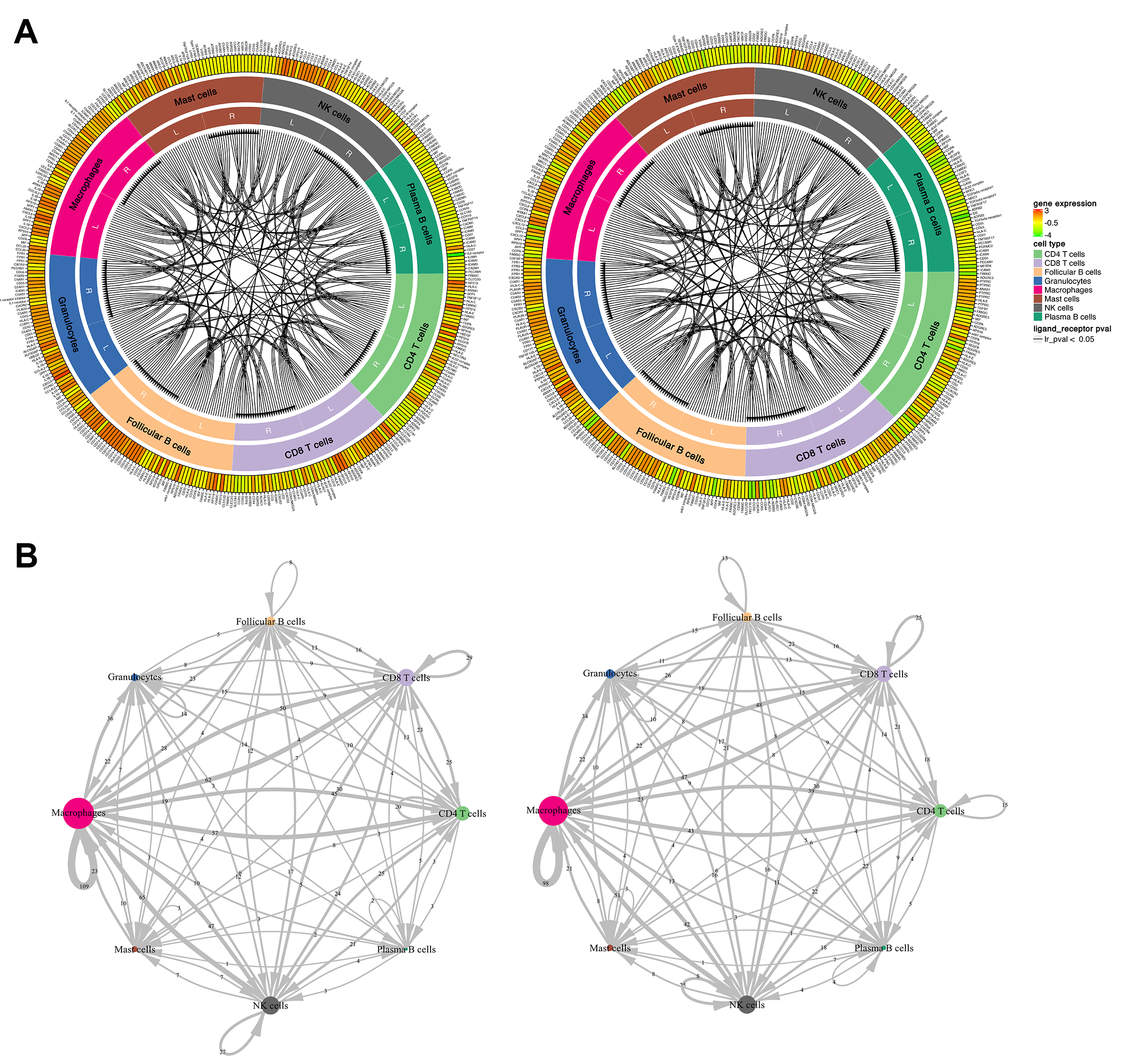

Supplement: Supplementary file 9 [file Image_9.TIF]
