# Supplementary material for: Single-Cell Sequencing of Immune Cells in Human Aortic Dissection Tissue Provides Insights Into Immune Cell Heterogeneity
Source: Front Cardiovasc Med. 2022 Mar 31;9:791875. doi: 10.3389/fcvm.2022.791875 (PMC9008490; doi:10.3389/fcvm.2022.791875)
Supplement: Supplementary file 10 [file Data_Sheet_1.docx]

**Supplementary Figure legends:**

**Supplementary Figure 1.** The distribution of the percentage of mitochondrial counts (left), nGene (middle), and the nUMI (right) before **(A)** and after **(B)** quality control. **(C)** Feature plot displaying expression level of genes encoding hemoglobin constituents (*HBA1* and *HBA2*) before quality control. UMI, unique molecular identifier.

**Supplementary Figure 2.** Heat map of the top 10 marker genes for the 16 cell clusters identified in all immune cells. The top 10 cluster-specific markers were selected based on the average Log (fold change).

**Supplementary Figure 3.** Heat map of the top 20 marker genes for the 5 myeloid cell clusters. The top 20 cluster-specific markers were selected based on the average Log (fold change).

**Supplementary Figure 4.** The KEGG pathway enrichment analysis of the 5 myeloid cell clusters via GSVA. KEGG, Kyoto Encyclopedia of Genes and Genomes; GSVA, Gene Set Variation Analysis.

**Supplementary Figure 5.** The GO-BP term enrichment analysis of 5 myeloid cell clusters via GSVA. GO-BP, Gene Ontology Biological Processes; GSVA, Gene Set Variation Analysis.

**Supplementary Figure 6.** Reclustering of T and NK cells. **(A)** A UMAP plot of 6,168 T and NK cells reclustered into 18 clusters. **(B)** Heat map of top 10 [by average Log (fold change)] marker genes in the 16 clusters of T and NK cells. **(C)** A UMAP plot representing canonical cell markers of different T cell subtypes and NK cells. **(D)** A UMAP plot of CD4+ T cells, CD8+ T cells and NK cells identified by their marker genes. NK cells, natural killer cells; UMAP, Uniform Manifold Approximation and Projection for Dimension Reduction.

**Supplementary Figure 7.** Heat map of the top 10 marker genes for the 6 CD4^+^ T cell clusters. The top 10 cluster-specific markers were selected based on the average Log (fold change).

**Supplementary Figure 8.** Reclustering of CD8^+^ T cells and NK cells. **(A)** Reclustering of CD8+ T cells into 5 clusters as shown by UMAP plot. **(B)** A UMAP plot showing CD8^+^ T cells from the AD and NA group, respectively. **(C)** Reclustering of NK cells into 5 clusters as shown by UMAP plot. **(D)** A UMAP plot showing NK cells from the AD and NA group, respectively. **(E)** Heat map of top 20 [by average Log (fold change)] marker genes in CD8^+^ T cells of AD and NA groups, respectively. **(F)** Heat map of top 20 [by average Log (fold change)] marker genes in NK cells of AD and NA groups, respectively. NK cells, natural killer cells; UMAP, Uniform Manifold Approximation and Projection for Dimension Reduction; AD, aortic dissection; NA, normal aorta.

**Supplementary Figure 9.** The dense network of cellular connection in the AD and NA group. **(A)** Circos diagram showing multiple cellular connection of immune cells in the AD (left) and NA (right) group. **(B)** The dense network of intercellular communication among immune cells in the AD (left) and NA (right) group. AD, aortic dissection; NA, normal aorta.

**Supplementary Table 1**: Estimated number of cells, mean reads per cell and mean genes per cell for sample

| Sample | Estimated number of cells | Mean reads per cell | Median genes per cell |
| --- | --- | --- | --- |
| AD1 | 3300 | 171453 | 1569 |
| AD2 | 6700 | 74811 | 1546 |
| AD3 | 6918 | 99013 | 1500 |
| Control1 | 9357 | 60273 | 1741 |
| Control2 | 17902 | 34855 | 1733 |

AD, aortic dissection.

**Supplementary Table 2**: Experimental conditions for the five-plex multiplexed immunofluorescence assay

| Staining order | Primary antibody | Clone | Vendor | Dilution | Incubation (hour) | TSA-Opal | Color |
| --- | --- | --- | --- | --- | --- | --- | --- |
| 1 | CD3 | CST#85061T | Cell Signaling | 1: 50 | 4 | Opal 570 | Yellow |
| 2 | CD68 | NB100-683 | Novus Biologicals | 1: 200 | 1 | Opal 520 | Green |
| 3 | CD20 | ab64088 | Abcam | 1: 200 | 1 | Opal 690 | Red |
| 4 | c-kit | ab32363 | Abcam | 1: 400 | 1 | Opal 480 | Cyan |
| 5 | CD66b | ab197678 | Abcam | 1: 100 | 2 | Opal 780 | Magenta |
